# Induction of IL-12p40 and type 1 immunity by *Toxoplasma gondii* in the absence of the TLR-MyD88 signaling cascade

**DOI:** 10.1371/journal.ppat.1009970

**Published:** 2021-10-01

**Authors:** Lindsay M. Snyder, Claire M. Doherty, Heather L. Mercer, Eric Y. Denkers

**Affiliations:** Center for Evolutionary and Theoretical Immunology and Department of Biology, University of New Mexico, Albuquerque, New Mexico, United States of America; University of Medicine and Dentistry of New Jersey, UNITED STATES

## Abstract

*Toxoplasma gondii* is an orally acquired pathogen that induces strong IFN-γ based immunity conferring protection but that can also be the cause of immunopathology. The response in mice is driven in part by well-characterized MyD88-dependent signaling pathways. Here we focus on induction of less well understood immune responses that do not involve this Toll-like receptor (TLR)/IL-1 family receptor adaptor molecule, in particular as they occur in the intestinal mucosa. Using eYFP-IL-12p40 reporter mice on an *MyD88*^*-/-*^ background, we identified dendritic cells, macrophages, and neutrophils as cellular sources of MyD88-independent IL-12 after peroral *T*. *gondii* infection. Infection-induced IL-12 was lower in the absence of MyD88, but was still clearly above noninfected levels. Overall, this carried through to the IFN-γ response, which while generally decreased was still remarkably robust in the absence of MyD88. In the latter mice, IL-12 was strictly required to induce type I immunity. Type 1 and type 3 innate lymphoid cells (ILC), CD4^+^ T cells, and CD8^+^ T cells each contributed to the IFN-γ pool. We report that ILC3 were expanded in infected *MyD88*^*-/-*^ mice relative to their *MyD88*^+/+^ counterparts, suggesting a compensatory response triggered by loss of MyD88. Furthermore, bacterial flagellin and *Toxoplasma* specific CD4^+^ T cell populations in the lamina propria expanded in response to infection in both WT and KO mice. Finally, we show that My88-independent IL-12 and T cell mediated IFN-γ production require the presence of the intestinal microbiota. Our results identify MyD88-independent intestinal immune pathways induced by *T*. *gondii* including myeloid cell derived IL-12 production, downstream type I immunity and IFN-γ production by ILC1, ILC3, and T lymphocytes. Collectively, our data reveal an underlying network of immune responses that do not involve signaling through MyD88.

## Author summary

*Toxoplasma gondii* is an apicomplexan parasite estimated to infect 30–50% of humans worldwide. The parasite normally establishes latency in brain and muscle tissue marked by persistent asymptomatic infection. *T*. *gondii* masterfully strikes a balance between eliciting strong, anti-parasite immunity while also persisting in the host. Although the murine host recognizes *Toxoplasma* profilin via MyD88 and Toll-like receptors 11/12, humans lack these receptors and MyD88 deficient patients retain resistance to *T*. *gondii* infection. Given these observations, it is important to identify MyD88 independent pathways of immunity. Using an oral infection mouse model, we identified cellular sources of IL-12 and IFN-γ, two cytokines that are essential for host resistance to this microbial pathogen. We determined how these responses are impacted by the presence and absence of MyD88 and the intestinal microbiota. Our data demonstrate that *T*. *gondii* triggers MyD88-independent innate and adaptive immunity in the intestinal mucosa that requires the presence of intestinal microbes. These pathways may be conserved among species and understanding how they work in rodents will likely help determine how humans recognize and respond to *T*. *gondii* infection.

## Introduction

*Toxoplasma gondii* is a widely distributed apicomplexan parasite estimated to be present in 30–50% of the world population, and is a leading cause of foodborne illness-related deaths in the United States [1]. Ingestion of contaminated food products or oocysts leads to acute infection, systemic dissemination of tachyzoites, and establishment of latent infection in brain and muscle tissue [2]. Although asymptomatic in most individuals, toxoplasmosis may emerge as a life-threating disease in immunocompromised patients and infection can lead to severe congenital defects and stillbirth during pregnancy [3,4].

As an orally acquired pathogen, the intestinal mucosa is the site of first encounter between *Toxoplasma* and the host immune system [5,6]. *Toxoplasma* actively infects the intestinal epithelium and cells within the underlying lamina propria (LP) [7]. It was long ago established that key to initiating the immune response to *T*. *gondii* is early production of IL-12 [8,9]. In the intestinal mucosa, IRF8-dependent CD103^+^CD11b^-^ dendritic cells (DC) are believed to be a primary subset of cells that produce IL-12 in response to *T*. *gondii* [10,11]. It has also been demonstrated that intestinal microbes act as natural adjuvants that support IL-12 production during *T*. *gondii* infection [12]. In turn, IL-12 drives induction of NK and T_H_1 cells that fuel protective immunity through production of IFN-γ [13]. Other cells such as neutrophils and innate lymphoid cells also supply IFN-γ during infection [14,15]. Inflammatory macrophages activated by IFN-γ are major effector cells involved in control of *Toxoplasma* [16,17]. While a strong Type 1 cytokine response is required for the host to survive infection, severe immunopathology involving proinflammatory cytokine overproduction, intestinal tissue destruction and bacterial translocation can result in the absence of regulation by cytokines such as IL-10 [18–21].

Intense interest has surrounded the question of how the innate immune system senses *Toxoplasma*. A decade-and-a-half ago, parasite profilin was discovered as a classical pathogen-associated molecular pattern (PAMP) molecule that was recognized by mouse Toll-like receptors (TLR) 11/12 [22–24]. Other studies have suggested a role for TLR7/8/9 in sensing parasite nucleic acids and for TLR2/4 in recognition of *Toxoplasma* glycolipids [25,26]. Previous studies have highlighted the important role of cell intrinsic MyD88 signaling in production of NK cell-derived IFN-γ and initiation of protective T cell immunity [27,28]. Overall, the profilin/TLR11 interaction has been regarded as the most prominent with regard to immune initiation. Nevertheless, profilin is unlikely to function as a universal PAMP insofar as many species, including humans, express neither TLR11 nor TLR12 in functional form [29]. More generally, human populations deficient in MyD88, a major signaling adaptor of TLR, retain resistance to all but a few pyogenic bacterial infections [30,31]. Thus, the PAMP-TLR-MyD88 axis appears to be much less important during immune recognition in humans and many other species. This realization has sparked new interest in identification of MyD88/TLR independent mechanisms of *Toxoplasma* recognition that may be relevant to human infection [32].

Despite the importance of the profilin-TLR11/12 axis in mice, it is also clear that other pathways of MyD88-independent immunity operate. For example, MyD88 knockout mice develop protective immunity to intraperitoneal and oral challenge following vaccination with *cps1-1*, an attenuated uracil auxotrophic strain of *T*. *gondii* [33]. It was also demonstrated that the same parasite strain elicits potent anti-tumor immunity independently of MyD88 [34]. In vitro, bone marrow-derived macrophages release IL-12 independently of MyD88, in part due to parasite GRA24 and GRA15_II_ [35–38]. Nevertheless, a detailed picture on the emergence of MyD88-independent immunity, particularly in the intestine where infection initiates, is lacking.

In this study, we establish that deletion of MyD88 reveals normally cryptic pathways of immunity in the intestinal mucosa that do not rely on TLR signaling. We analyzed innate and adaptive immune responses generated in response to peroral inoculation with *T*. *gondii* including myeloid cell-mediated production of IL-12, T cell and innate lymphoid cell derived IFN-γ, and the generation of antigen-specific CD4^+^ T cells. We found that while IL-12 responses were lower in the absence of MyD88, an infection-induced response nonetheless persisted. Our results indicate MyD88-independent IL-12 production is both necessary and sufficient to drive IFN-γ production following infection. We also found that microbiota-specific and *T*. *gondii-*specific CD4^+^ T cells are generated in the LP regardless of MyD88 expression. Furthermore, the MyD88-independent IL-12 and T cell mediated IFN-γ responses observed in the small intestine were dependent upon the presence of the intestinal microbiota.

## Results

### MyD88-independent anti-*Toxoplasma gondii* immunity is triggered in the small intestine

*Toxoplasma* elicits MyD88-dependent and MyD88-independent immunity during oral infection [33,39–41], but the extent of these responses in the intestinal mucosa is relatively unexplored. Here, we found that oral inoculation with *T*. *gondii* induced robust systemic IFN-γ production in both MyD88 wild-type (WT) and MyD88 knockout (KO) mice, although KO levels were approximately 50% of that observed in WT serum (S1A Fig). We also employed quantitative PCR to determine parasite levels in WT and KO mice (S1B Fig). To obtain values for total parasite levels in each location, whole tissue was homogenized then an aliquot was used for downstream processing. Parasite levels were significantly higher in the spleen (approximately 3-fold increase) and most notably the liver (approximately 30-fold increase) in MyD88-deficient mice (S1B Fig). Nevertheless, levels in intestinal mucosal tissues were not significantly different in WT vs. KO animals. Previously, using immunohistochemistry we reported increased parasite numbers in the small intestine and Peyer’s patches of *MyD88*^*-/-*^ mice, which we did not see here. Possible reasons for the difference are considered below (see Discussion).

**Fig 1:**
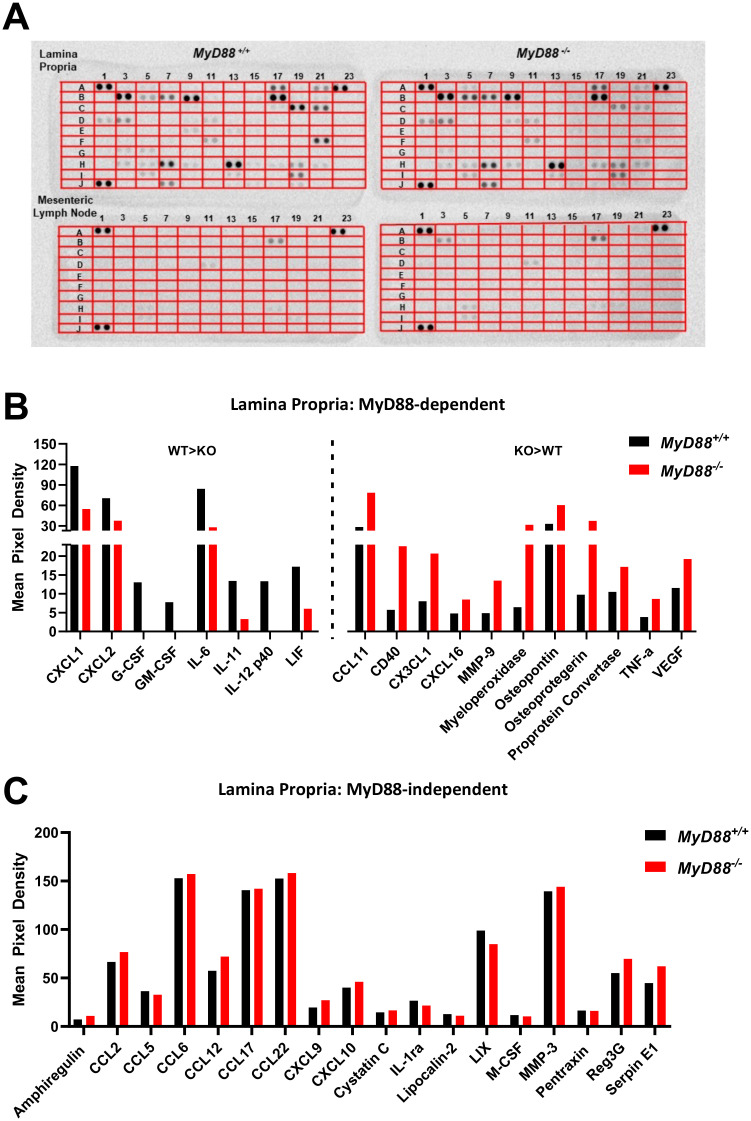
*T*. *gondii* induces both MyD88-dependent and MyD88-independent cytokine and chemokine responses in the intestinal mucosa. Lamina propria (LP) cells from *MyD88*^*+/+*^ and *MyD88*^*-/-*^ mice were isolated at day 7 post-oral infection with 40 ME49 cysts and cultured *ex vivo* without further stimulation for 72 hrs. (A) The resulting supernatants were collected, pooled, and analyzed for cytokines and chemokines using a commercial proteome array. Mean pixel densities of MyD88-dependent LP responses (defined as 1.7-fold change or greater between mouse strains) were plotted (B) and compared to MyD88-independent responses (C). Only cytokines and chemokines with mean pixel densities above background are displayed. Supernatants were pooled from cells derived from 4 mice/group.

Although *MyD88*^*-/-*^ mice display a defect in systemic type I immunity we sought to characterize the local intestinal, MyD88-independent immune response generated in response to peroral *T*. *gondii* infection. To broadly characterize the intestinal immune response to *Toxoplasma*, we employed a commercial multiplex protein array containing 111 immune mediators to gain insight into the influence of MyD88 during infection in the intestinal mucosa. MyD88 WT and KO mice were inoculated with cysts of the Type II ME49 strain, then lamina propria (LP) and mesenteric lymph node (MLN) cells were harvested one week later and cultured *ex vivo* for determination of cytokines, chemokines and other immune-related factors. Analysis of the resulting supernatants revealed that the LP compartment was more immunologically active than the MLN in both *MyD88*^*+/+*^ and *MyD88*^*-/-*^ mice (Fig 1A; a key indicating the coordinates of each protein on the proteome array is shown in S2 Fig). In the LP compartment, we identified a subset of chemokines and cytokines that were MyD88-dependent (Fig 1B). For example, CXCL1, CXCL2, IL-6, and IL-12 were more highly expressed by WT LP cells (Fig 1B, left side) whereas CCL11, CX3CL1, CD40 were more highly expressed by KO LP cells (Fig 1B, right side). Nevertheless, a larger subset of inflammatory mediators was expressed independently of MyD88 (Fig 1C). Prominent in this category were the key chemokines CCL6, CCL12, CCL17 and CCL22. While the MLN compartment was relatively silent compared to the LP, we nonetheless identified several proteins including CCL6 (Fig 1A; coordinate B3) that were differentially expressed by WT and KO cells, and at least one chemokine (CCL22; Fig 1A; coordinate B17) that was expressed regardless of MyD88 expression. These data show that MyD88-independent as well as MyD88-dependent mucosal immunity is present during *T*. *gondii* infection, and that the LP is far more immunologically active than the MLN compartment.

### Myeloid cell derived IL-12p40 is produced in the absence of MyD88 during intestinal *Toxoplasma* infection

IL-12 is essential in initiating a protective T_H_1 response against *T*. *gondii* [8,42]. Our proteome array failed to detect IL-12 in the absence of MyD88 (Fig 1A and 1B), but it was possible this was an issue of assay sensitivity. To further search for MyD88-independent cellular sources of intestinal IL-12, we began by examining responses in noninfected LP cells exposed to *in vitro* stimulation with *Toxoplasma*. Naïve *MyD88*^*+/+*^ LP cells exposed to sonicated tachyzoite antigen (STAg) or infected *ex vivo* with Type I (RH) or Type II (PTG) tachyzoites produced low amounts of IL-12p40, whereas stimulation of *MyD88*^*-/-*^ cells failed to elicit IL-12p40 production above background levels (S3A Fig). Our lab and others have observed that *T*. *gondii* dense granule secretory protein GRA24 induces MyD88-independent bone marrow derived macrophage IL-12 production via phosphorylation and activation of p38 MAPK [36,37,43,44]. To determine if we could detect this pathway in the intestinal mucosa, we infected WT and KO LP cells with type I parasite strain-derived *cps1-1* and *cps1-1*Δ*gra24* tachyzoites *in vitro* and measured IL-12 production. In both WT and KO cells, presence or absence of GRA24 had no impact on IL-12 production (S3B Fig). *In vitro* infection of *MyD88*^*-/-*^ LP cells with the different parasite strains failed to elicit IL-12. We also probed the supernatants for cytokines and chemokines using the proteome array described in Fig 1. A pixel density cutoff >2 was employed to graph chemokines and cytokines above background levels. The proteome signatures between *cps1-1* and *cps1-1*Δ*gra24* infected LP cells were remarkably similar, although in this assay IL-12p40 and TNFRSF11B appeared to be slightly lower in the absence of GRA24 (S3C Fig).

Loss of the MyD88 adaptor protein had no effect on LP cell yields or lymphocyte frequencies, although we observed a non-significant decrease in the frequency of CD8^+^ T cells (S4A and S4B Fig). When frequencies were corrected to cell numbers, we obtained a significant reduction in both CD4^+^ and CD8^+^ T cell numbers in the MyD88 KO LP (S4C Fig). In contrast, myeloid cell frequencies and numbers in the LP were not dependent upon the expression of MyD88 (S4D and S4E Fig).

**Fig 2:**
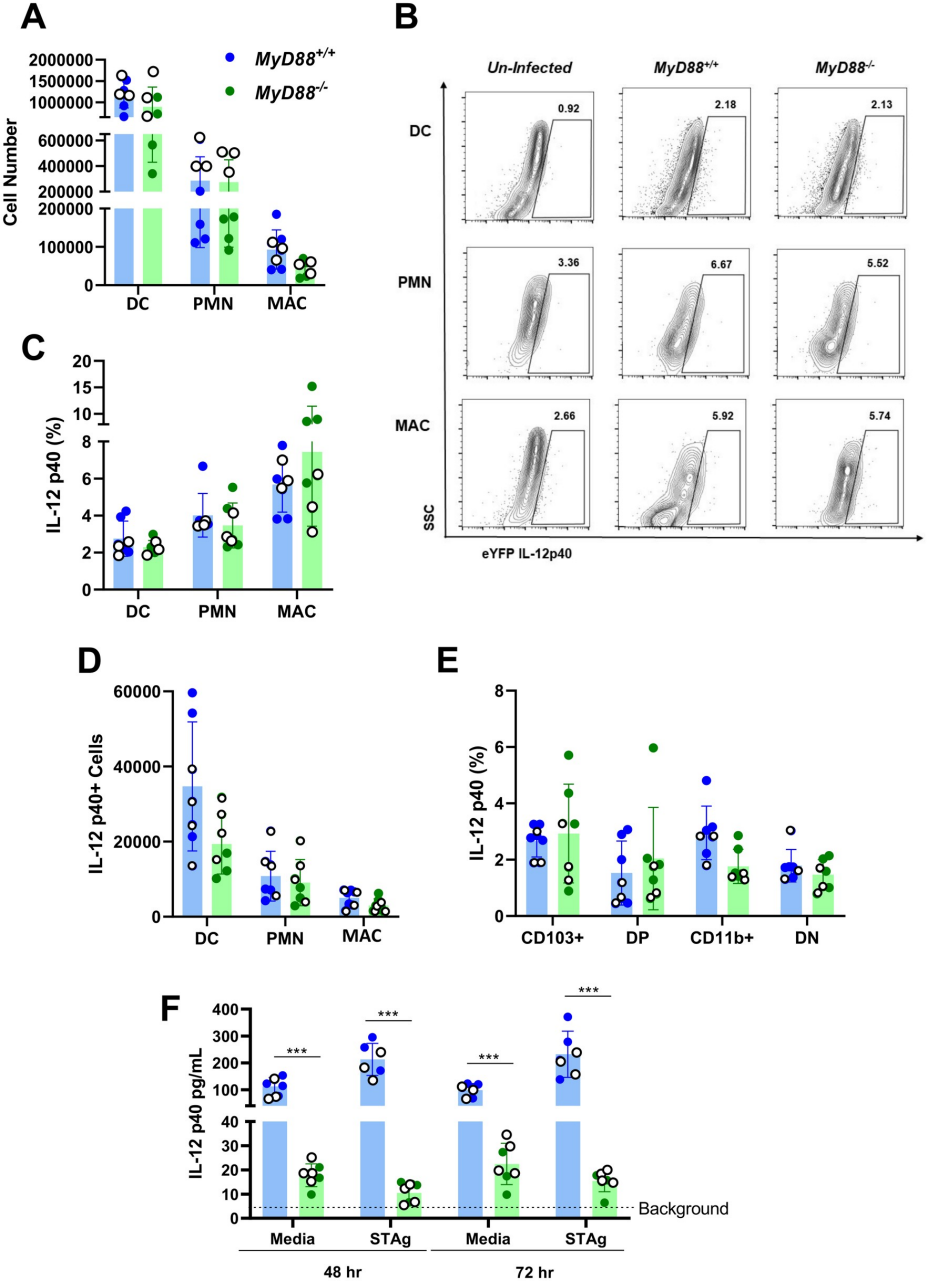
Lamina propria dendritic cells, neutrophils, and macrophages produce IL-12p40 in the absence of MyD88 during *T*. *gondii* infection. *MyD88*^+/+^ and *MyD88*^-/-^ *IL-12p40 eYFP* mice were orally inoculated with 40 ME49 cysts. At day 7 post-infection, small intestinal LP cells were collected for analysis. (A) Dendritic cell (DC; MHCII^+^ CD11c^+^ F4/80^-^), neutrophil (PMN; Ly6G^+^), and macrophage (MAC; F4/80^+^) cell numbers as determined by flow cytometry. (B) IL-12p40 expression in myeloid subsets from IL-12p40 reporter mice on *MyD88*^*+/+*^ and *MyD88*^*-/-*^ backgrounds. *Un-infected* shows background levels in a representative *MyD88*^*+/+*^ *IL-12p40 eYFP* mouse. Numbers indicate the percent of each cell type falling within the indicated quadrant. (C) IL-12p40^+^ cell frequencies and (D) cell numbers determined in DC, PMN and MAC. (E) Expression of IL-12p40 in DC (MHCII^+^ CD11c^+^ F4/80^-^) subsets based upon expression of CD103 and CD11b. DP, double positive for CD103 and CD11b. DN, double-negative for CD103 and CD11b. (F) *Ex vivo* IL-12p40 secretion by LP cells from infected mice cultured for 48 or 72 hours. Media, untreated; STAg, treated with soluble tachyzoite antigen. Dashed line indicates background IL-12p40 levels secreted by noninfected LP cells from WT and KO mice. Values are the means ± SEM of two independent experiments where each symbol represents a single mouse. White dots indicate data from experiment 1; color-filled dots indicate data from an independent experiment. Unpaired *t* test was employed to analyze the data, where * p<0.05.

To determine IL-12p40 producing cell populations during *in vivo* infection of MyD88 KO mice we backcrossed *MyD88*^*+/+*^ *IL-12p40-eYFP* reporter mice with *MyD88*^*-/-*^ animals to generate *MyD88*^*-/-*^ *IL-12p40-eYFP* reporter mice [36]. We collected LP cells from infected reporter mice for flow cytometric analysis. Myeloid cells including dendritic cells (DC; MHCII^+^CD11c^+^F4/80^-^), macrophages (F4/80^+^), and neutrophils (Ly6G^+^) were defined as shown in S5 Fig. DC, neutrophil, and macrophage cell numbers were overall similar between *MyD88*^*+/+*^ and *MyD88*^*-/-*^ mice at day 7 post-infection (Fig 2A). We next analyzed IL-12p40-eYFP expression in WT and KO myeloid cells (Fig 2B) and found that as expected, IL-12p40-eYFP expression was induced by *T*. *gondii* infection. Furthermore, IL-12p40^+^ myeloid cell frequencies (Fig 2C) and cell numbers (Fig 2D) were comparable between genotypes. IL-12p40 mean fluorescent intensities (MFI) were similar for each cell subset analyzed, regardless of MyD88 expression. DC were further sub-gated into CD103^+^, CD103^+^CD11b^+^ (DP), CD11b^+^, and CD103^-^ CD11b- (DN) subsets [45]. Loss of MyD88 expression had no effect on the frequencies of IL-12p40^+^CD103^+^, DP, CD11b^+^, or DN DC cells (Fig 2E). LP cells from infected mice were also cultured *ex vivo* to quantify IL-12p40 secretion. In this case, while we detected MyD88-independent IL-12, levels were clearly lower than those derived from WT LP cells (Fig 2F). The reason for the discrepancy between the flow cytometry (IL-12 levels equivalent in WT and KO mice) and the bulk LP cultures (IL-12 present, but lower in KO cultures) is unclear. However, we note that the flow cytometry data represents an instantaneous snapshot of eYFP expression, whereas the LP culture data represent an *in vitro* accumulation of IL-12 secreted over 48 to 72 hours. Regardless, both the flow cytometry and ELISA data demonstrate that *Toxoplasma* infection elicits IL-12 production that does not involve the MyD88 signaling adaptor.

MLN cells were also collected from infected mice and similar experiments were performed to determine IL-12p40 producing cells in the presence and absence of MyD88. Total numbers of MLN DC, neutrophils, and macrophages were similar between WT and KO mice (S6A Fig). IL-12-eYFP expression was analyzed within each myeloid cell subset and the frequency and number of IL-12p40^+^ DC, neutrophils, and macrophages was found to be similar between *MyD88*^*+/+*^ and *MyD88*^*-/-*^ mice (S6B–S6D Fig). Interestingly, the most striking IL-12p40 responses were in the macrophage populations where approximately 20% of cells were positive for the cytokine in both WT and KO mice (S6B and S6C Fig). Nevertheless, when corrected for cell number (S6D Fig), DC accounted for the majority of IL-12 expressing cells in both WT and KO populations. When DC were gated into CD103^+^, DP, CD11b^+^ and DN subsets, presence or absence of MyD88 had no effect on IL-12, and the major IL-12-positive population was the CD103^+^ subset (S6E Fig). After culturing MLN cells *ex vivo*, we quantified IL-12p40 protein and found that KO cells produced IL-12, although at a lower level compared to WT cells (S6F Fig). *T*. *gondii-*induced IL-12 production was similar between MLN and LP cells (Fig 2F and S6F Fig); however, we note that eYFP-IL-12 flow staining, in particular for macrophages, was more robust in the MLN. Together, these results demonstrate that peroral *T*. *gondii* infection induces MyD88-independent mucosal immunity and measurable IL-12 production by DC, neutrophils, and macrophages.

### *T*. *gondii* infection induces type 1 immunity in *MyD88*^*-/-*^ mice

**Fig 3:**
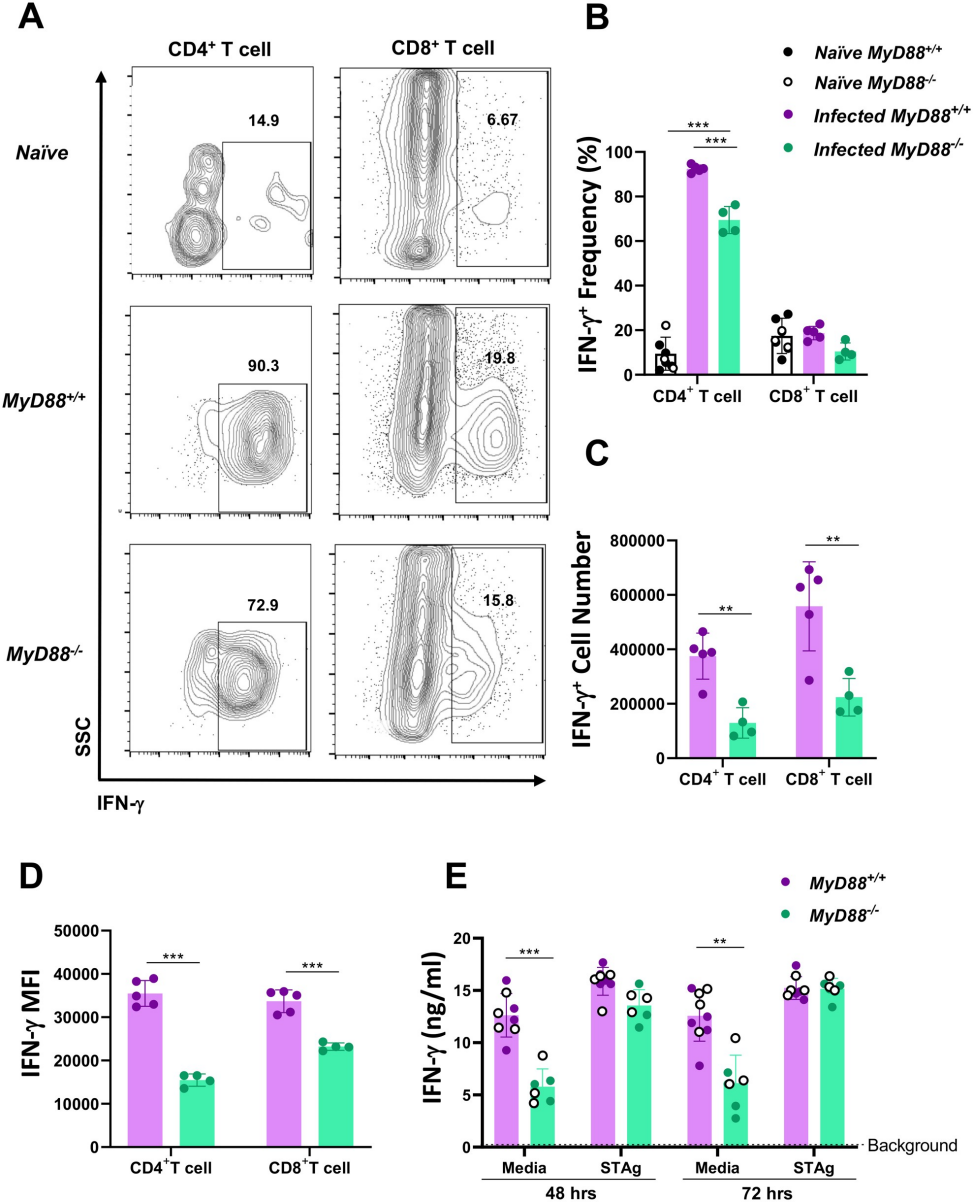
*T*. *gondii* triggers a vigorous MyD88-independent IFN-γ response in the small intestine. Lamina propria cells were collected from naïve and day 7 infected *MyD88*^*+/+*^ and *MyD88*^*-/-*^ mice, and T lymphocyte IFN-γ production was assessed by flow cytometry. (A) Representative contour plots showing T cell IFN-γ expression in MyD88 WT and KO mice. Numbers indicate the percentage of cells falling within gates. *Naïve* shows the responses of noninfected WT and KO mice. (B) The frequency of IFN-γ^+^ cells and (C) total number of IFN-γ^+^ T cells over multiple mice. (D) Mean fluorescence intensity (MFI) of IFN-γ positive CD4^+^ and CD8^+^ T cell populations in the LP. (E) IFN-γ production during *ex vivo* culture of LP cells cultured in medium. Background levels of IFN-γ released by cells from noninfected WT and KO mice are indicated by the dashed line. The data shown in E are pooled from two independent experiments, where white dots indicate data obtained from experiment 1, and color-filled dots indicate data obtained from a second independent experiment. Flow cytometry data is representative and was repeated three times with similar results. An unpaired Student’s *t* test was used to determine significance of data in C-E, and one-way ANOVA with Tukey multiple comparisons post-test was employed to analyze data in B. In this figure, * p<0.05 **p<0.01 ***p<0.001.

To determine if IL-12p40 produced in the absence MyD88 (Fig 2 and S6 Fig) was sufficient to drive intestinal type I immunity, we collected LP cells from infected WT and KO mice and analyzed T cell IFN-γ production by flow cytometry. In Day 7-infected mice, CD4^+^ T cell frequencies were modestly reduced in the LP compartment (S7A Fig), while no significant change was detected in the MLN (S7B Fig). *T*. *gondii* infection induced a dramatic expansion of IFN-γ^+^ CD4^+^ T cells in both *MyD88*^*+/+*^ and *MyD88*^*-/-*^ mice (Fig 3A). However, the frequency and most notably number of T_H_1 cells were reduced in infected KO mice compared to WT mice (Fig 3B and 3C). Expression of IFN-γ in CD8^+^ T lymphocytes was not significantly impacted by infection in either WT or KO cells (Fig 3A and 3B). Nevertheless, like CD4^+^ T cells, the total number of IFN-γ-positive CD8^+^ T cells was reduced in the absence of MyD88 (Fig 3C). Furthermore, IFN-γ MFI values were significantly reduced in MyD88 deficient CD4^+^ and CD8^+^ T cells (Fig 3D). *Ex vivo* culture of *MyD88*^*+/+*^ and *MyD88*^*-/-*^ LP cells revealed robust levels of IFN-γ protein secretion (Fig 3E). Although KO levels did not reach WT levels in the absence of STAg stimulation, following stimulation with parasite antigen, *MyD88*^*-/-*^ cells retained the ability to produce comparable amounts of IFN-γ (Fig 3E). The data reveal that although IFN-γ^+^ T cell numbers are reduced in the KO LP, robust production of IFN-γ remains intact in the absence of MyD88.

MLN cells were also collected from mice at day 7 post-infection and T cell IFN-γ was assessed. IFN-γ expressing CD4^+^ and CD8^+^ T cells from naïve and infected mice were quantitated (S8A and S8B Fig). *T*. *gondii* infection induced a robust T_H_1 response, although loss of the MyD88 adaptor protein resulted in a significantly reduced frequencies of IFN-γ^+^ CD4^+^ T cells (S8B Fig). IFN-γ^+^ CD8^+^ T cell frequencies were similar between WT and KO mice, but there was a non-statistically significant trend for increased IFN-γ expression by CD8^+^ T cells from infected mice compared to naïve mice (S8A and S8B Fig). Unlike in the LP compartment, IFN-γ MFI in the MLN were similar between WT and KO T cells (S8C Fig). To quantitate release of IFN-γ, we cultured cells *ex vivo* and found that STAg stimulated WT cells produced higher levels of IFN-γ compared to stimulated KO cells (S8D Fig). In sum, although IFN-γ^+^ T cell numbers and secreted IFN-γ levels were reduced in MyD88 KO mice, *T*. *gondii* still induced a relatively robust type I immune response in the gut mucosa.

### Microbiota and *Toxoplasma* specific CD4^+^ T cells contribute to MyD88-independent immunity

**Fig 4:**
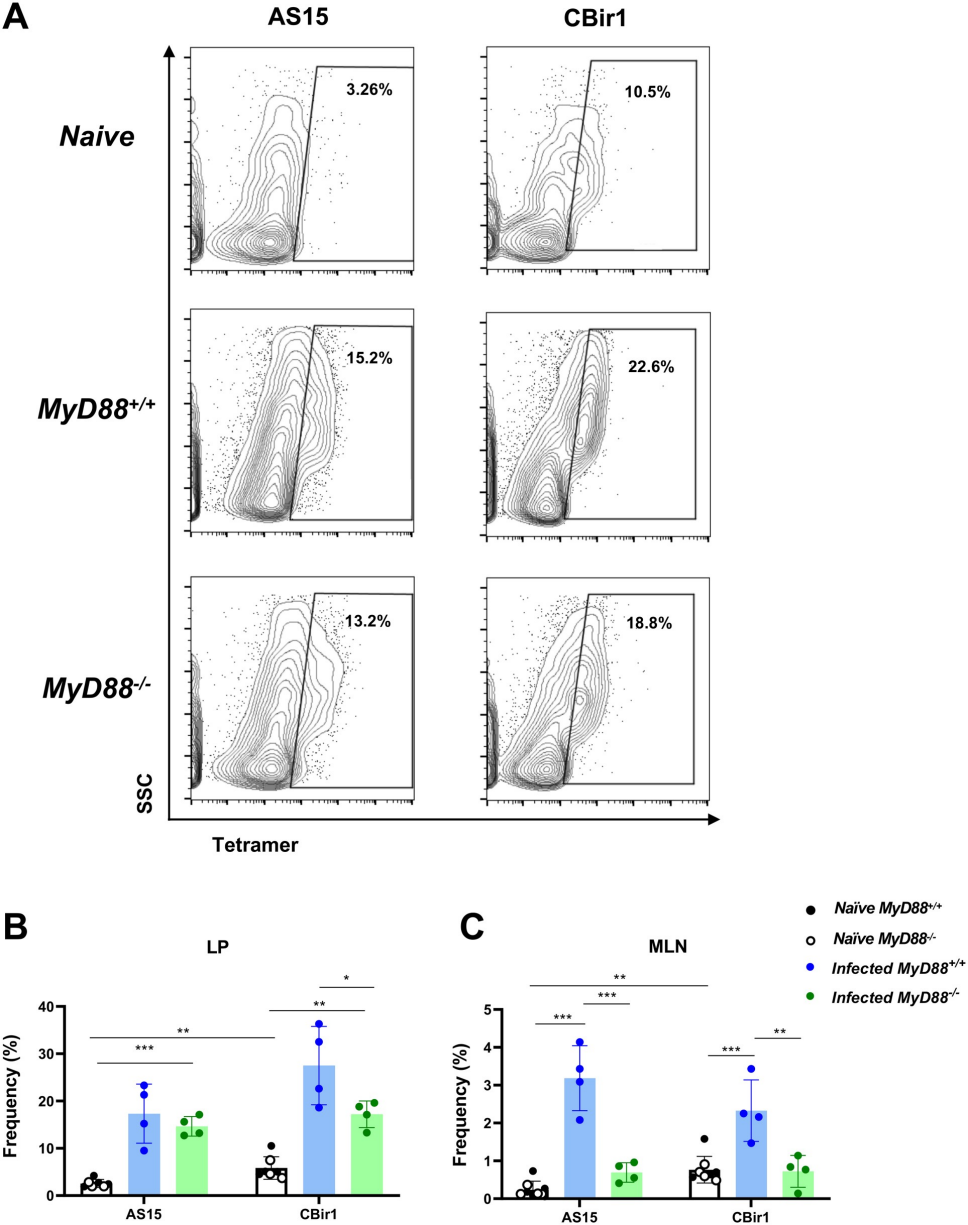
Differential MyD88 requirements for expansion of *T*. *gondii* and microbiota specific CD4^+^ T cells in the lamina propria and mesenteric lymph node. Small intestine LP cells and MLN cells were isolated from *MyD88*^*+/+*^ and *MyD88*^*-/-*^ mice at day 7 post-infection. (A) *T*. *gondii* and microbiota specific CD4^+^ T cells quantified by staining with an APC labeled MHCII tetramer loaded with *Toxoplasma* peptide epitope AS15 and PE labeled MHCII tetramer loaded with flagellin peptide epitope CBir1. Tetramer-specific CD4^+^ T cell frequencies in the small intestine LP (B) and MLN (C). Each symbol represents a single mouse, and the experiment was repeated independently with similar results. Unpaired Student’s *t* test (C) where *p<0.05 **p<0.01.

*T*. *gondii* infection induces translocation of intestinal microbes into the LP compartment [19,20]. Consequently, parasite specific and microbiota specific T cell responses are generated [12,46]. To quantitate antigen specific CD4^+^ T cell responses, we used MHCII tetramers loaded with a *T*. *gondii* peptide derived from AS15 (AVEIHRPVPGTAPPS) [47] and MHCII tetramers loaded with a *Clostridium*-related flagellin peptide (YSNANILSQ; an immunodominant microbiota antigen) [46,48]. The gating strategy to examine CD4^+^ T cells is shown in S9A Fig. Representative flow gating of tetramer positive cells showed that infection induced a substantial increase in *T*. *gondii* and flagellin specific CD4^+^ T cell frequencies in the LP compartment (Fig 4A). To confirm the specificity of the MHCII tetramers, staining was assessed in the CD8^+^ T cell gate (S9B Fig). Tetramer staining in the LP CD8^+^ T cell gates were well below what was observed in CD4^+^ T cells (S9B and S9C Fig). Within the LP, parasite specific CD4^+^ T cells were strongly induced by infection in both WT and KO mice (Fig 4B). Although flagellin tetramer^+^ CD4^+^ T cells were readily detectable in naïve mice, *T*. *gondii* infection induced an expansion in these microbiota specific T cells in *MyD88*^*+/+*^ and *MyD88*^*-/-*^ mice (Fig 4B). The prior observation that CD4^+^ T cell frequencies were reduced in the MyD88 deficient LP (S7A Fig) indicates that antigen specific CD4^+^ T cell numbers would also be reduced in the KO small intestine. Even so, it is noteworthy that parasite and flagellin specific CD4^+^ T cells expand during *Toxoplasma* infection even in the absence of MyD88. In the MLN, antigen specific T cell frequencies were overall almost 10-fold lower than that observed in the LP. Additionally, the generation of *T*. *gondii* and microbiota specific CD4^+^ T cells was highly impaired in KO mesenteric lymph nodes compared to WT (Fig 4C). These data suggest that the expansion of *T*. *gondii* and microbiota specific CD4^+^ T cell responses in the LP functions largely independently of MyD88, whereas antigen specific T cell responses in the MLN are reliant upon the MyD88 signaling pathway.

### Small intestinal ILC1 and ILC3 produce IFN-γ in response to *T*. *gondii* infection

To determine how innate lymphoid cells (ILC) contribute to intestinal anti-*Toxoplasma* immunity, we analyzed their function in the LP compartment. First, we examined steady state levels in noninfected animals. Conventional ILC were designated as lineage negative (CD3^-^, Ly6C^-^, Ly6G^-^, CD11b^-^, B220^-^, TER-119^-^) CD90^+^ CD127^+^ cells (S10A Fig). Interestingly, we detected a substantial population of unconventional ILC that were lineage negative, CD90^+^, CD127^-^. Within the CD127^+^ ILC gate, ILC1, ILC2, and ILC3 were further designated as T-bet^+^, GATA3^+^, and RORγt^+^ respectively (S10B Fig). Similarly, T-bet^+^ ILC1, GATA3^+^ ILC2, and RORγt^+^ ILC3 frequencies were quantified within the CD127^-^ ILC gate (S10C Fig.) Naïve WT and KO mice had similar frequencies and cell numbers of CD127^+^ and CD127^-^ ILC1, ILC2, and ILC3 (S10D–S10G Fig).

**Fig 5:**
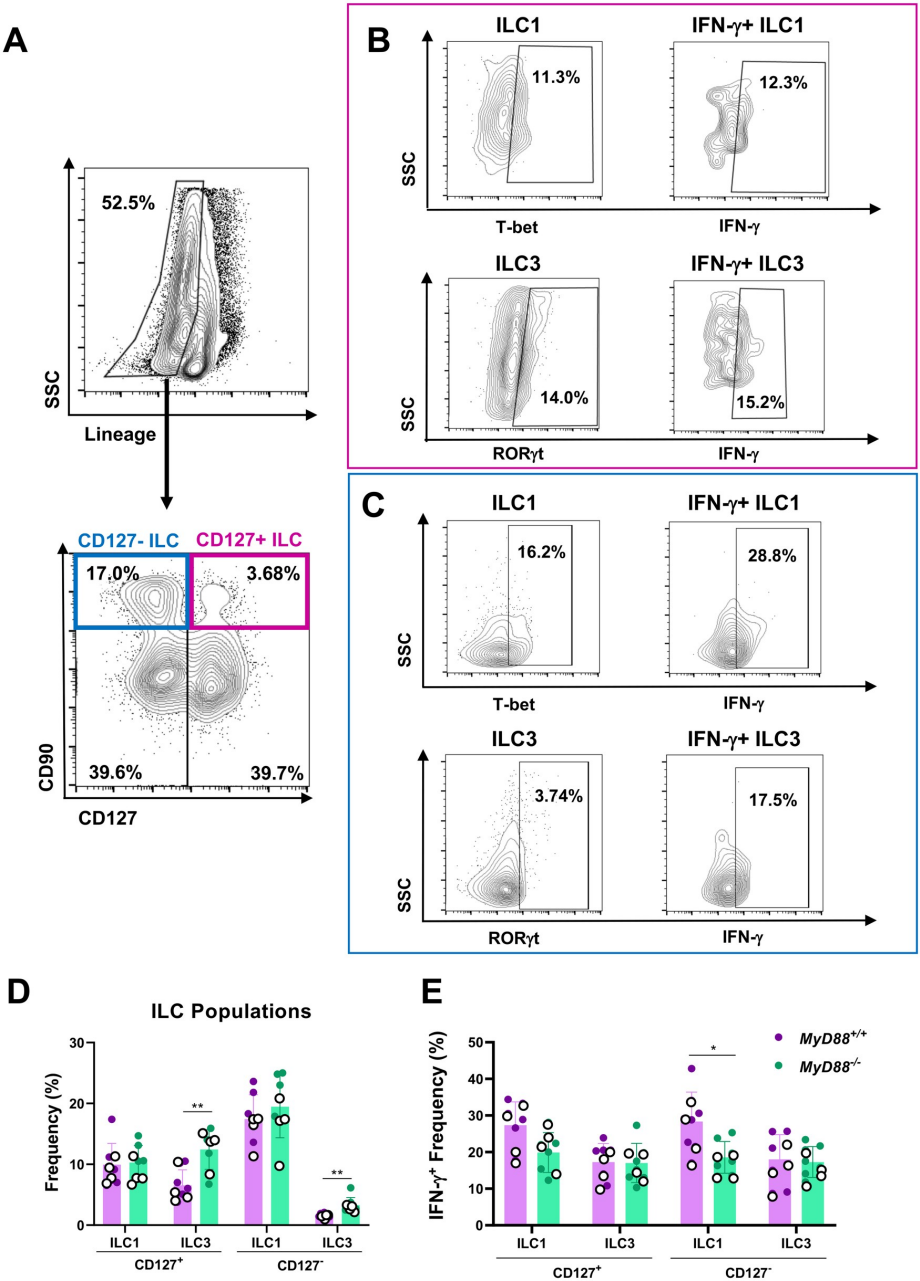
MyD88 contributes to the IFN-γ-producing innate lymphoid cell compartment in the small intestine. LP cells were isolated from infected MyD88 WT and KO mice at day 7 post-infection and ILC activity was determined by flow cytometry. (A) Flow gating scheme to identify CD127^+^ and CD127^-^ ILC. Numbers indicate the percent of cells falling within each gate. (B) CD127^+^ staining for T-bet (above) and RORγt (below). Within each population, IFN-γ staining is shown in the scattergrams on the right. (C) In the same fashion, markers of ILC1 and ILC3, and expression of IFN-γ was assessed in the CD127^-^ population. (D) Frequency of ILC populations and (E) frequency of frequency of IFN-γ^+^ producing ILC1 and ILC3 were determined from gating shown in A-C. Values are the means ± SEM of two independent experiments where each symbol represents a single mouse and open and closed symbols dots delineate between experiments. Unpaired Student’s *t* test (D, E) was used to determine statistical significance where **p<0.01.

Intracellular staining for IFN-γ was used to assess the CD127^+^ and CD127^-^ ILC1 and ILC3 contribution to type 1 immunity during *Toxoplasma* infection (Fig 5A–5C). At day 7 post-infection, CD127^+^ and CD127^-^ ILC3 frequencies were elevated in KO mice compared to WT controls (Fig 5D). When we examined IFN-γ in these populations, we found that CD127^-^ IFN-γ^+^ ILC1 frequencies were reduced in *MyD88*^*-/-*^ mice, although the frequency of IFN-γ^+^ ILC3 remained similar between genotypes (Fig 5E). Taken together, these data suggest that ILC1 and ILC3 both contribute to MyD88-independent type I immunity, and that the ILC3 subset may be expanded in the MyD88 deficient mucosa to compensate for diminished ILC1 function.

### IL-12 is required to drive MyD88-independent type 1 immunity during oral *T*. *gondii* infection

**Fig 6:**
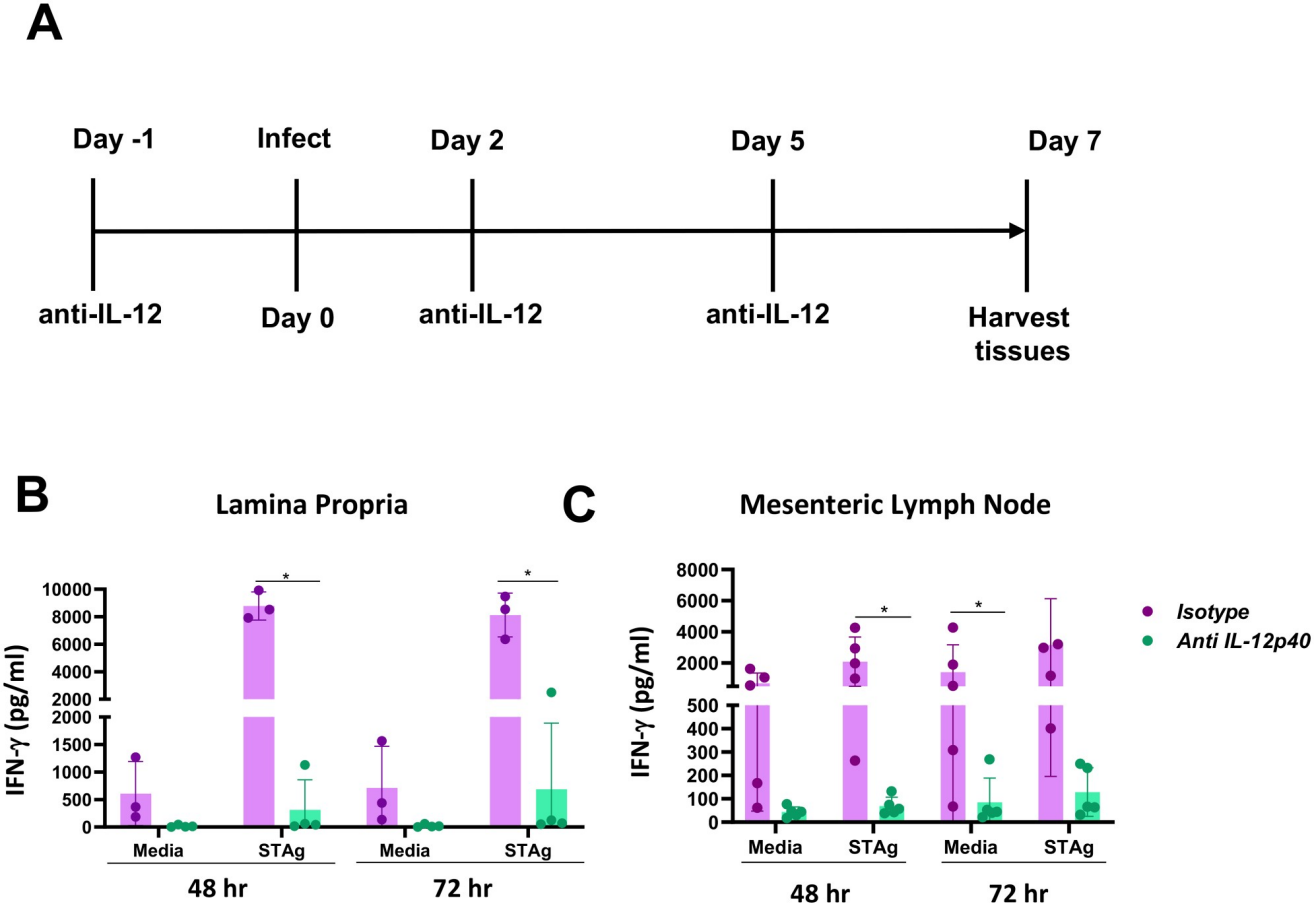
MyD88-independent IFN-γ response requires IL-12. (A) *MyD88*^*-/*-^ mice were subjected to injections of IL-12p40 neutralizing antibody before and after infection. (B) LP cells and (C) MLN cells were prepared at day 7 post-infection and cultured with media alone or stimulated with STAg for the indicated times. Supernatants were collected and IFN-γ was quantified by ELISA. An independent experiment was performed with similar results. An unpaired Student’s *t* test was used to analyze the data where * p<0.05.

In WT mice, IL-12 is required for the induction of protective, type I immunity during *T*. *gondii* infection [8,42]. Infected MyD88 KO mice produced significantly less IL-12 during *in vitro* culture relative to WT mice, yet, even though reduced, the ensuing T_H_1 response was vigorous in both strains. This observation led us to determine whether MyD88-independent T_H_1 immunity was dependent upon IL-12p40. To assess the role of IL-12 in inducing MyD88-independent production of IFN-γ, *MyD88*^*-/-*^ mice were treated with IL-12p40 depleting antibodies prior to and during infection (Fig 6A). Cells harvested from isotype control treated MyD88 KO mice produced large amounts of IFN-γ (Fig 6B and 6C). In striking contrast, IFN-γ production was reduced to near undetectable levels in LP and MLN cells isolated from IL-12p40 depleted mice (Fig 6B and 6C). Therefore, we conclude that reduced IL-12 produced in *MyD88*^*-/-*^ mice is both necessary and sufficient to drive a type I immune response during *T*. *gondii* infection.

Although IL-12 was required to induce MyD88-independent, intestinal IFN-γ (Fig 6B and 6C), it was possible that other mucosal cytokines play a role in amplifying type I immunity. For example, TNF-α has been shown to play an important role in NK cell-mediated production of IFN-γ in response to *T*. *gondii* infection [49,50]. The proteome array in Fig 1 also indicated that TNF-α levels were elevated in *MyD88*^*-/-*^ LP supernatants compared to *MyD88*^*+/+*^ LP supernatants (Fig 1B). To determine the role of TNF-α in inducing type I immunity in the absence of MyD88, KO mice were injected with anti-TNF-α monoclonal antibody before and during peroral *T*. *gondii* infection as detailed in the schematic (S11A Fig). In contrast to the IL-12 depletion (Fig 6), we did not detect an effect of anti-TNF-α antibody on production of IL-12p40 or IFN-γ upon in vitro culture (S11B Fig). These data suggest that compared to IL-12, TNF-α plays a minor role in anti-*Toxoplasma*, type I immunity generated in the absence of MyD88.

### The intestinal microbiota plays a major role in driving MyD88-independent immunity against *T*. *gondii*

**Fig 7:**
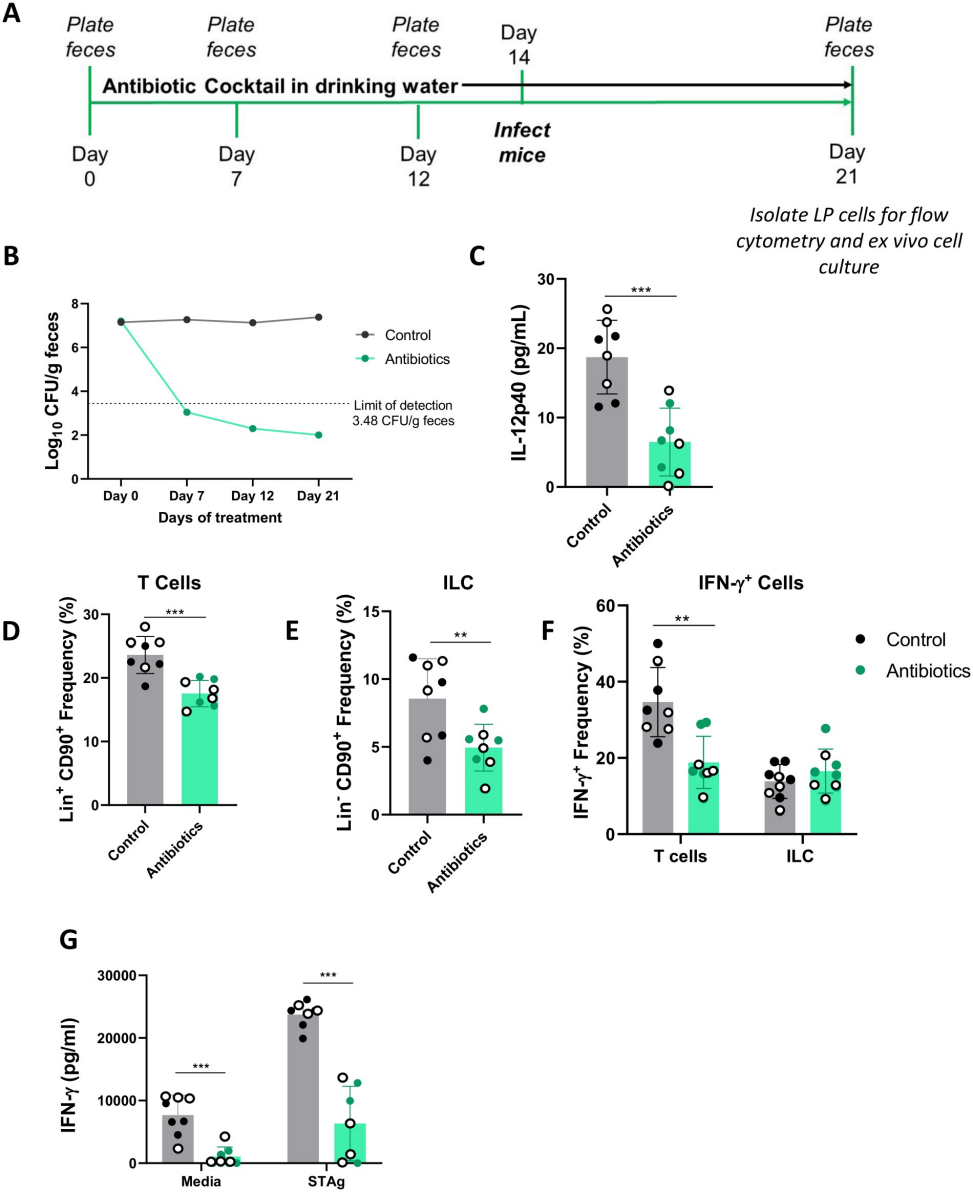
Intestinal microbiota is required for MyD88-independent mucosal immunity against *T*. *gondii*. (A) *MyD88*^*-/-*^ mice were treated with antibiotics two weeks prior to and throughout infection. Feces and tissues were collected at time points indicated. (B) Fecal CFU were determined weekly throughout the infection. At day 7 post-infection, tissues were harvested, and LP cells were cultured *ex vivo*. Supernatants were harvested after 72 hr and (C) IL-12 was quantified. (D) T cell (Lineage^+^, CD90^+^) and (E) ILC (Lineage^-^, CD90^+^) frequencies in the LP were determined by flow cytometry. (F) Within the T cell and ILC gates, IFN-γ^+^ frequencies were quantified. (G) IFN-γ levels from *ex vivo* cell cultures were determined by ELISA. Values are the means ± SEM of two independent experiments. Open and closed symbols indicate animals from independent experiments. An unpaired Student’s *t* test was used to analyze the data where **p<0.01 *** p<0.001.

It has been established that intestinal microbes breach the intestinal barrier during peroral *T*. *gondii* infection [20]. Furthermore, translocating microbes act as adjuvants to drive IL-12 production, and microbiota (flagellin) specific CD4^+^ T cell populations expand and contribute to type I immunity [12,46]. Therefore, we sought to determine the role of the intestinal microbiota in MyD88-independent immunity in the intestinal mucosa. MyD88 KO mice were treated with an antibiotic cocktail in their drinking water one week prior to, and throughout infection (Fig 7A). Within one week, culturable fecal CFU dropped below the level of plating detection (Fig 7B). Antibiotic-treated and control mice were orally inoculated and lamina propria cells were harvested at day 7 post-infection for *ex vivo* culture. Compared to LP cells from control mice, LP cells from antibiotic mice secreted significantly less IL-12 (Fig 7C). To characterize the type I immune response, LP T cell and ILC populations were evaluated by flow cytometry. Lineage^+^ CD90^+^ T cell frequencies (Fig 7D) and Lineage^-^ CD90^+^ ILC frequencies (Fig 7E) in the small intestine were significantly reduced by antibiotic treatment. T cell function was also negatively impacted because IFN-γ producing T cell frequencies were reduced in antibiotic treated mice (Fig 7F). Interestingly, IFN-γ+ ILC frequencies were similar between control and antibiotic treated mice (Fig 7F). In *ex vivo* cell cultures, IFN-γ production by LP cells was dramatically reduced by antibiotic treatment. We conclude that the intestinal microbiota is required for generating both IL-12 and IFN-γ in response to *Toxoplasma*. Both T cell and ILC populations are decreased in microflora-depleted animals, and additionally T cell IFN-γ production is negatively impacted by loss of the microbiota.

## Discussion

The gastrointestinal tract is a portal of infection for many pathogens, and host survival hinges on rapid detection of these microbes and initiation of protective innate and adaptive immunity while maintaining tolerance to the gut microbiota [51–53]. Although many of these pathways of immune induction rely upon TLR signaling and the MyD88 adaptor protein, it is clear that other important pathways exist in both humans and mice [30,33,54–58]. In this study, we use a mouse model to identify intestinal anti-*Toxoplasma* immunity generated in the absence of MyD88. In *MyD88*^*-/-*^ mice, peroral inoculation of *T*. *gondii* cysts induced dendritic cell, macrophage, and neutrophil derived IL-12. Due to the major role DC play in initiating systemic anti-*Toxoplasma* immunity [11,59] and the observation that DC were the most numerous IL-12^+^ myeloid cell in the small intestine LP, we speculate that DC contribute largely to the MyD88-independent, intestinal IL-12 pool. The observed infection-induced IL-12 was necessary to drive type I immunity. In the absence of MyD88, ILC1, ILC3, CD4^+^, and CD8^+^ T cells produced IFN-γ, a cytokine that is central in the protective immune response to *T*. *gondii* [60–62]. Both *Toxoplasma* and microbiota specific CD4^+^ T cell populations underwent infection-induced expansion in the *MyD88*^*+/+*^ and *MyD88*^*-/-*^ lamina propria.

In MyD88 KO mice, we observed increased parasite burden in the spleen and liver. Nevertheless, *Toxoplasma* levels throughout the small intestine were similar between WT and KO mice. This differs from our published immunohistochemistry study where we reported increased parasite burden in *MyD88*^*-/-*^ LP and Peyer’s patches [33]. Since *T*. *gondii* establishes discrete foci of infection throughout the gut, it is possible that the histology data were not completely representative of the entire length of the intestine [63]. The current qPCR employing DNA prepared from whole tissues suggests that overall parasite burdens are similar within the intestine regardless of MyD88 expression. This indicates that the impact of MyD88 loss may be most profound after dissemination from the gut. Nevertheless, we also note that the PCR analysis was performed at a single time point (namely, that at which the immunological studies were performed). Thus, it is possible that at later time points intestinal parasite burdens between WT and KO mouse strains would diverge. Finally, we note that our previous study was performed well over a decade ago and it is possible that genetic drift of the parasite is a contributing factor.

In the absence of MyD88 signaling, *T*. *gondii* stimulated intestinal IL-12 and a type I immune response, although overall the response was weaker than that occurring in WT mice. In the present study, we did not directly address the protective capability of immune cells generated in the absence of MyD88. However, we previously found that *MyD88*^*-/-*^ mice vaccinated with the attenuated, uracil auxotroph strain *cps1-1* develop protective immunity to oral challenge with ME49 [33]. Additionally, we recently published data indicating that the parasite dense granule protein GRA24 plays a role in driving MyD88-independent IL-12 leading to protective immunity in *cps1-1*-vaccinated *MyD88*^*-/-*^ mice [36]. Together these studies show that functional adaptive immunity can be generated in the absence of MyD88, although an optimal response requires this signaling adaptor. Future studies will be directed towards determining the ability of different cell subsets of the mucosal immune system to confer MyD88-independent protection.

It has been established that intestinal microbes translocate into the lamina propria and act as natural adjuvants during *Toxoplasma* infection [12,20,64]. Interestingly, we observed that depletion of the intestinal microbiota severely impacted MyD88-independent immune responses to peroral *T*. *gondii* infection. Bacterial-derived LPS can signal independently of MyD88 through TLR4 and the TRIF/TRAM pathway to activate NFκB and type I interferons in an MyD88-independent manner [65,66]. It is plausible that PAMPs derived from translocating intestinal microbes signal through TLR4 to induce adaptive immunity in the absence of MyD88. Another possibility is that microflora derived molecules, including peptidoglycan or muramyl dipeptide, signal through NOD-like receptors to promote MyD88-independent triggering of immunity [67–69]. The adjuvant effect of the microbiota is most likely strongest in the intestinal mucosa and we posit that this explains the robust IFN-γ based immune response and parasite control we observed in this tissue.

Some *Toxoplasma* secreted molecules are known to bypass the TLR-MyD88 pathway and directly hijack downstream host signaling to trigger IL-12. GRA15 is a polymorphic parasite dense granule protein injected into the host cell cytoplasm from within the parasitophorous vacuole. The Type II allele of GRA15 induces NFκB activation, IL-12 production, and subsequent type I immunity [37,38,40]. Similarly, GRA24 is a dense granule protein that induces parasite strain-independent p38 MAPK autophosphorylation and IL-12 production [36,43]. Here, we found that GRA24 plays a limited role in directly inducing IL-12 production by *MyD88*^*+/+*^ and *MyD88*^*-/-*^ LP cells since both WT and GRA24 KO parasites stimulated equivalent amounts of LP IL-12 during *in vitro* culture. The finding that during intraperitoneal infection GRA24 promotes a protective immune response and that absence of GRA24 leads to increased parasite number in the peritoneal cavity suggests that this molecule may have a more prominent role as parasites disseminate beyond the intestinal mucosa [36,37,43].

Other sources of parasite-triggered MyD88-independent immunity might include factors resulting from inflammasome activation. In response to *in vitro T*. *gondii* infection, NOD-like receptors including NLRP1, NLRP3, and NALP1 assemble resulting in the release of IL-1β and IL-18 [39,70,71]. However, arguing against *Toxoplasma* directly inducing IL-12 within an infected cell as would be predicted for inflammasome assembly or activation mediated by GRA proteins, the majority of IL-12 production by LP DC derives from *Toxoplasma* negative populations [10]. Other studies also show that mucosal DC are not major targets of invasion during oral infection [63]. Therefore, it is likely that early IL-12 is triggered indirectly by host factors rather than molecules produced by the parasite itself. In this regard, host alarmins S100A11 and IL-33 have been implicated in monocyte recruitment and aggravated ileitis respectively during *T*. *gondii* infection, although their role in IL-12-driven immunity is not clear [41,72].

While the response was clearly reduced, the IL-12 produced by MyD88 deficient LP and MLN cells was necessary and sufficient to trigger a robust type I intestinal immune response. Antigen-specific LP CD4^+^ T cells in MyD88 KO mice also expanded similar to frequencies observed in the WT small intestine, in particular with respect to AS15, the *Toxoplasma*-specific tetramer. Interestingly, *Toxoplasma* and microbiota specific CD4^+^ T cell frequencies overall were lower in the MLN compared to LP compartment. Furthermore, the MLN compartment displayed a greater dependence upon MyD88, insofar as the frequency of tetramer-positive KO CD4^+^ T cell populations was close to the background levels seen with CD8^+^ T cells (Fig 4 and S9C Fig).

In line with this observation, overall IFN-γ^+^ CD4^+^ T cell frequencies were between 70–90% in the LP and between 8–12% in the MLN in both WT and KO mice. The proteome array (Fig 1) also indicated that the MLN compartment was far less immunologically active than the LP regardless of MyD88 expression. It seems likely that additional signals in the gut, whether microbial- or host-derived, help drive both MyD88-dependent and MyD88-independent immunity and support expansion of antigen-specific T cells in the gut. Evidence that this is the case comes from other studies showing that *TLR11*^*-/-*^ mice retain resistance to *Toxoplasma* following oral infection, and when the mice are depleted of gut microbiota they exhibit a diminished Th1 response and increased host susceptibility [12]. This contrasts with intraperitoneal infection where immunity and resistance display a greater TLR11 and MyD88 component [12]. Thus, in the peritoneal cavity expansion of antigen specific CD4^+^ T cells and generation of protective immunity appears to require TLR/MyD88 signaling in the absence of contributions from the intestinal environment.

We observed that ILC1 and ILC3 contributed to MyD88-independent type I immunity. Although ILC are classically defined by expression of CD127 (IL-7R), we detected a large population of lineage negative, CD127^-^, CD90^hi^ ILC that co-expressed T-bet and RORγt and stained positive for IFN-γ. Other groups have reported the existence of such CD127^-^ ILC in the lung and liver [73,74]. In addition, it is possible that ILC may internalize and degrade or shed CD127 in response to IL-7, similar to what has been observed in T cells [75,76]. Further studies on the importance of IL-7R signaling in ILC in the context of *Toxoplasma* infection are warranted. In addition to ILC mediated production of IFN-γ, we observed an increase in ILC3 frequencies in infected *MyD88*^*-/-*^ mice. The role of intestinal mucosa type 3 ILC in anti-*Toxoplasma* immunity remains largely unexplored, although there is data indicating a role for ILC1 in early IFN-γ production and limiting parasite replication in the peritoneal cavity [77]. ILC3 frequencies have been shown to decrease during infection, although these cells may also play a role in limiting pathology and T cell responses [78]. The reduction of ILC1 and concomitant expansion of ILC3 in the MyD88 deficient LP that we report here may be due to the presence or absence of certain cytokines. ILC3 differentiation is driven by the transcription factor RORγt along with polarizing cytokines including IL-2, IL-23, and IL-1β [79]. Additionally, retinoic acid derived from CD103^+^ DCs can also act to induce ILC3 differentiation [80]. It is possible that changes in the cytokine milieu or retinoic acid metabolism could drive the shift from ILC1 to ILC3 in the MyD88 deficient small intestine. We are currently further examining the functional significance of IFN-γ-producing ILC3 during *Toxoplasma* infection.

We observed reduced steady state IFN-γ protein secretion the LP and MLN cultures from infected KO cells compared to WT mice. Yet, *MyD88*^*-/-*^ cells retained the ability to respond to STAg stimulation and produced levels of IFN-γ comparable to WT cells. This observed type I immune response was entirely abrogated with IL-12 depletion and antibiotic-mediated depletion of the intestinal microbiota. Together, these data indicate that the microbiota and/or *T*. *gondii* induce sufficient amounts of IL-12 to drive a type I immune response and expansion of antigen specific T cells in the MyD88 deficient small intestine. Further elucidation of MyD88-independent pathways of mucosal immunity may shed light on how humans sense infection and mount immune responses to *Toxoplasma* and other microbial pathogens in the absence of MyD88.

## Materials and methods

### Ethics statement

All experiments were performed in strict accordance with the recommendations set forth by the National Institutes of Health *Guide for the Care and Use of Laboratory Animals* (8^th^ Edition). Protocols were approved by the Institutional Animal Care and Use Committee at the University of New Mexico (Animal Welfare Assurance Number A4023-01). All efforts were made to minimize animal suffering and distress over the course of studies performed.

### Mice

B6.129-*Il12b*^*tm1*.*1Lky*^/J (IL12p40-eYFP, Stock # 006412) and B6.129P2(SJL)-*Myd88*^*tm1*.*1Defr*^/J (*MyD88*^*-/-*^, Stock # 009088) mice were purchased from The Jackson Laboratory (Bar Harbor, ME). IL12p40-eYFP mice were bred with *MyD88*^-/-^ mice, and the resulting heterozygous F1 progeny were crossed to obtain F2 *MyD88*^*-/-*^ *IL12p40-eYFP* animals [36]. Homozygous F2 *MyD88*^*+/+*^ *IL12p40-eYFP* and *MyD88*^*-/-*^ *IL12p40-eYFP* mice were maintained in a breeding colony in the Department of Biology Animal Research Facility at the University of New Mexico. 4-6-week-old female B6NTac (C57BL/6) and Swiss Webster mice were purchased from Taconic (Rensselaer, NY).

### Parasites and infections

8 to 12-week-old Swiss Webster mice were intraperitoneal infected with 20 cysts of the type II *T*. *gondii* strain ME49 to establish a chronically infected mouse colony. After establishment of chronic infection (3–4 weeks post-infection), mouse brains were collected and homogenized to obtain a suspension of ME49 cysts. Infections in experimental mice were initiated by orally inoculating with 40 ME49 cysts in 200 μl PBS. Type I (RH) and type II (PTG) tachyzoites were maintained *in vitro* by successive infection of human foreskin fibroblasts (ATCC, Manassas, VA, USA).

The design and construction of Type I uracil auxotroph *T*. *gondii* parasite strains *ΔompdcΔup* (*cps1-1*) and *ΔompdcΔupΔgra24* (*cps1-1*:*Δgra24)* has been previously described [34,81]. *cps1-1* and *cps1-1*:*Δgra24* tachyzoites were maintained *in vitro* by serial passage in human foreskin fibroblast monolayers in media supplemented with 300 μM exogenous uracil using previously described methods [36]. Parasites were passaged once a month into media without supplemental uracil to ensure that parasites had not reverted and retained dependence upon exogenous uracil.

### PCR detection of *Toxoplasma gondii*

Whole tissues were harvested from naïve and infected mice and homogenized in 2–5 mL of PBS. Then 500 μl of homogenate was centrifuged to pellet both host cells and any extracellular tachyzoites. Genomic DNA was extracted from the pellet using the DNeasy Blood and Tissue Kit (Qiagen Inc. Valencia, CA). To determine systemic parasite burdens, quantitative PCR was performed targeting the conserved *Toxoplasma* B1 gene and the murine argininosuccinate lyase (ASL) gene as previously described [82,83]. Briefly, 5 μM primer solutions containing the forward and reverse primers for B1 (forward 5’-GGA-GGA-CTG-GCA-ACC-TGG-TGT-CG-3’, reverse 5’-TTG-TTT-CAC-CCG-GAC-CGT-TTA-GCA-3’) and the forward and reverse primers for ASL (forward 5’-TCT-TCG-TTA-GCT-GGC-AAC-TCA-CCT-3’, reverse 5’-ATG-ACC-CAG-CAG-CTA-AGC-AGA-TCA-3’) were prepared. Real-time qPCR reactions were prepared as follows: 5 μl of template, 2.5 μl of 5 μm B1 or ASL primer solution, 10 μl SYBR green (Bio-Rad), and 2.5 μl molecular biology grade water. The reactions were carried out using a Bio-Rad CFX96 Real Time System C1000 Touch thermal cycler. The number of B1 and ASL gene copies were quantified using a standard curve (Bio-Rad CFX manager version 3 software). Tissue parasite burdens were reported as the number of *Toxoplasma* genomes per host genome.

### Isolation of primary leukocytes

Small intestines were collected and processed as previously described to obtain lamina propria cells [84]. Briefly, small intestines were washed, then fat tissue and Peyer’s patches were removed. The cleaned tissue was cut longitudinally and trimmed into 1 cm pieces. The small intestine pieces were incubated twice for 20 minutes at 37°C in calcium/magnesium-free Hank’s Balanced Salt Solution (HBSS), 10 mM EDTA (VWR, Radnor, PA, USA), and 1 mM dithiothreitol (Sigma Aldrich, St. Louis, MO, USA) to release intraepithelial lymphocytes. A subsequent incubation for one hour at 37°C in Dulbecco’s Modified Eagle’s Medium (DMEM; Gibco, Grand Island, NY, USA) containing 300 U/mL collagenase (Worthington Biochemical, Lakewood, NJ, USA) was performed to release lamina propria cells. To purify the lamina propria cell population, cells were centrifuged without terminal braking in a discontinuous Percoll (Sigma Aldrich) gradient (40%/80% diluted with DMEM). The cells at the interface were collected and used for downstream applications. Mesenteric lymph nodes were collected, homogenized to release leukocytes, and passed through a 40 μm filter (VWR) prior to use in downstream applications.

### Cell culture and ELISA

Single-cell suspensions were cultured *ex vivo* at 2 x 10^6^ cells/mL in complete DMEM (cDMEM) consisting of 10% bovine growth serum (HyClone, Logan, UT, USA), 1% non-essential amino acids (Thermo Fisher Scientific, Waltham, MA, USA), 30 mM HEPES (Thermo Fisher Scientific), 1 mM sodium pyruvate (Thermo Fisher Scientific), 100 U/mL penicillin + 0.1 mg/mL streptomycin (Thermo Fisher Scientific), and 50 μM 2-mercaptoethanol (Sigma Aldrich). Cells were cultured in media alone, or stimulated with soluble tachyzoite antigen (STAg, 50 μg/mL). STAg was prepared by sonicating RH strain tachyzoites at 0°C in the presence of protease inhibitors, followed by centrifugation at 10,000 x g. Supernatant was collected and dialyzed into PBS and stored in aliquots at -80°C. The resulting cell culture supernatants were collected 6 to 24 hours post-plating. IL-12p40 and IFN-γ levels were quantified by ELISA following the manufacturer’s recommendations (Invitrogen, Waltham, MA, USA).

### Proteome array

Lamina propria and mesenteric lymph node cells were collected from naïve and infected *MyD88*^*+/+*^ and *MyD88*^*-/-*^ *IL12-p40 eYFP* mice. The cells were cultured *ex vivo* in cDMEM and supernatants were collected after 72 hours. Cytokines and chemokines were quantified simultaneously in samples using the Proteome Profiler Mouse XL Cytokine Array kit following the manufacturer’s protocol (R&D Systems, Minneapolis, MN). Briefly, the proteome array blots were hydrated and blocked, incubated with the supernatant samples overnight, and probed with biotinylated detection antibodies and streptavidin-horseradish peroxidase. After application of the substrate (3,3’,5,5’-Tetramethylbenzidine), blots were visualized on a Chemi Touch Imaging System (Bio-Rad), and Image J software was used to quantitate the mean pixel intensities of each cytokine and chemokine using background subtraction of control spots containing no cytokine/chemokine antibody.

### Flow cytometry

To study IFN-γ production, cells (1 x 10^6^) were stimulated in 1 mL FACS buffer (1% BGS /30 mM NaN_3_ in PBS) containing 50 ng/mL PMA (Sigma Aldrich), 5 μg/mL ionomycin (Alfa Aesar, Tewksbury, MA, USA), and 10 μg/mL Brefeldin A (Biolegend, San Diego, CA, USA, Catalog #420601) for 4 hours at 37°C. Single cell suspensions were subsequently incubated with Live/Dead Fixable Aqua (Thermo Fisher Scientific) for five minutes in PBS to stain dead cells. After quenching in FACS buffer and a subsequent wash, cell surface markers were labeled with primary antibodies including anti-MHCII Alexa Fluor 647 (Biolegend, Catalog #107618), anti- CD11c eFluor610 (Thermo Fisher Scientific Catalog #61-0114-82), anti-F4/80 Brilliant Violet 711 (Biolegend, Catalog #123147), anti-Ly6G PE/Cy7 (Biolegend, Catalog #127618), anti-Lineage cocktail Pacific Blue (Biolegend, Catalog #133310, anti-CD3, anti-GR1, anti-CD11b, anti-B220, anti-TER-119), anti-CD90 PE (Biolegend, Catalog #105307), anti-CD127 APC/Cy7 (Biolegend, Catalog #135039), anti-CD4 PerCP/Cy5.5 (Biolegend, Catalog #100434), anti-CD8a PE/Cy7 (Biolegend, Catalog #100722), or anti-TCRb APC/Cy7 (Biolegend, Catalog #109220) for 20 minutes at 4°C in FACS buffer. APC conjugated MHCII tetramer loaded with *T*. *gondii* antigen (CD4Ag28m peptide AVEIHRPVPGTAPPS), and PE conjugated MHCII tetramer loaded with flagellin peptide (CBir1 peptide YSNANILSQ; courtesy of the NIH Tetramer Core, Atlanta, GA, USA) were incubated with freshly isolated cells for 30 minutes at 37°C in DMEM prior to surface marker staining as described above [46,47]. After surface marker staining, cells were rinsed, fixed in 3.7% paraformaldehyde in PBS (15 minutes at room temperature), washed, and re-suspended in FACS buffer for analysis. For intracellular and nuclear staining, cells were rinsed and incubated overnight in 1 mL of Fixation/ Permeabilization buffer from the FoxP3/Transcription Factor Staining Kit (eBioscience, Waltham, MA, USA). The following day, samples were rinsed with 2 mL of permeabilization buffer (Foxp3Transcription Factor Staining Kit) prior to staining intracellular and nuclear proteins in permeabilization buffer for one hour at 4°C. Intracellular and nuclear markers were labeled using primary antibodies including anti-T-bet APC (Biolegend, Catalog #644814), anti-RORγt Brilliant Violet 650 (BD Biosciences, San Jose, CA, USA, Catalog #564722), anti- GATA3 Alexa Fluor 488 (Biolegend, Catalog #653807), or anti-IFN-γ (Biolegend, Catalog #505808). After one hour, samples were washed and re-suspended in FACS buffer prior to analysis. Samples were run on an Attune NXT, 5 laser flow cytometer (ThermoFisher Scientific) and the data were analyzed using FlowJo v.10 software (FlowJo).

### *In vivo* antibody depletions

Mice were intraperitoneally injected with 0.5 mg anti-IL-12p40 monoclonal antibody (BioXCell, Lebanon, NH, USA, Catalog #BE0051), 0.5 mg anti-TNF-α monoclonal antibody (BioXCell, Catalog # BE0058), or 0.5 mg rat gamma globulin (Jackson Immunoresearch, West Grove, PA, USA, Catalog #012000002) one day prior to infection. On day 0, mice were orally inoculated with 40 ME49 cysts. Subsequent anti-IL-12p40 and isotype antibody injections occurred on day 2 and day 5 post-infection. Anti-TNF-α and isotype antibody injections occurred on day 1, 3, and 5 post-infection. On day 7 post-infection, cells were isolated and cultured *ex vivo* with STAg (50 μg/ml). Supernatants were collected after 48 and/or 72 hours, and cytokine production was measured via ELISA (Invitrogen).

### Intestinal microbiota depletion

*MyD88*^*-/-*^ mice were given *ad libitum* access to antibiotic cocktail in their drinking water for two weeks prior to, and throughout infection. The antibiotic cocktail was prepared by dissolving vancomycin hydrochloride (0.5 g/L, VWR, Catalog #97062–554), ampicillin (1g/L, Aldon Corp SE, VWR Catalog #470233–552), metronidazole (1g/L, Acros Organics, VWR Catalog #200013–382), neomycin sulfate (1g/L, Enzo Life Sciences, VWR Catalog #89149–866) and sucrose (10%, VWR, Catalog #BDH9308-500G) in de-ionized water [85]. Water was changed weekly and protected from direct light exposure.

### Determination of fecal CFU

Fresh feces were collected from control and antibiotic-treated *MyD88*^*-/-*^ mice and diluted 1 to 10 with sterile PBS. The fecal slurry was serially diluted with PBS and 100 μl of various dilutions was plated on LB agar (BD Biosciences, Catalog #240110) plates. Plates were incubated overnight at 37°C and colonies enumerated the following day.

### Statistical analyses

Statistical analyses were performed using GraphPad Prism v.8 (GraphPad, La Jolla, CA). Two-tailed Student’s *t*-tests were used to compare normally distributed data with only two groups. A Student’s *t-*test with Welch’s correction was also used to compare data sets with unequal variances and a Mann Whitney test was used to compare data sets that were not normally distributed. A one-way ANOVA with Tukey multiple comparisons post-test was used to compare three or more normally distributed groups. A confidence interval of 95% (α = 0.05) was used as the cut off to denote significant changes between groups.

## Supporting information

S1 Fig Impact of MyD88 on systemic IFN-γ and organ-specific *Toxoplasma* levels. *MyD88*^*+/+*^ and *MyD88*^*-/-*^ mice were orally inoculated with 40 ME49 cysts. (A) One-week after infection, serum IFN-γ levels were measured by ELISA (n = 6-8/genotype). Naïve WT and KO serum IFN-γ (n = 3-4/genotype) was not detected in the ELISA (ND). (B) Duodenum, jejunum, ileum, spleen, liver, and MLN tissues were also collected from mice at day 7 post-infection. Parasite burden was quantified using tissues from mice that were orally infected with 40 cysts. Shown is a representative experiment of 4 performed. An unpaired Student’s *t* test was used to compare infected WT and KO mice where *p<0.05 **p<0.01 ***p<0.001.
(TIF)

S2 Fig Coordinates of chemokines and cytokines in proteome array. (TIF)

S3 Fig Cytokine profiles induced by *in vitro* infection of *MyD88*^*+/+*^ and *MyD88*^*-/-*^ LP cells from noninfected mice. (A) Naïve LP cells were cultured *ex vivo* with STAg or infected with a type I strain (RH) or type II strain (PTG) of *T*. *gondii* at the indicated MOI. Supernatants were collected after 72 hrs and IL-12p40 was quantified by ELISA. Values are the means ± SEM of three independent experiments. (B) Similarly, supernatants from *cps1-1* and *cps1-1*Δ*gra24* infected *MyD88*^*+/+*^ and *MyD88*^*-/-*^ LP cells were collected to quantify IL-12p40 production by ELISA. (C) Naïve WT LP cells were infected with *cps1-1* or *cps1-1*Δ*gra24* tachyzoites (MOI 1:1) and supernatants were harvested at 72 hours to analyze secreted immune factors using a mouse cytokine/chemokine proteome array. An unpaired Student’s *t* test was used to compare infected WT and KO responses where ***p<0.001.
(TIF)

S4 Fig Baseline leukocyte levels in naïve *MyD88*^*+/+*^ and *MyD88*^*-/-*^ lamina propria cell populations. Naïve mice were euthanized for tissue collection. (A) Total LP cell counts. Flow cytometry was used to determine frequencies of lymphocytes and myeloid cells (B and D, respectively), and total numbers of lymphocytes and myeloid cells were subsequently calculated (C and E, respectively). Values are the means ± SEM of three independent experiments with a total of n = 9 mice/genotype. Unpaired Student’s *t* test used to compare genotypes where *p<0.05.
(TIF)

S5 Fig Representative flow gating of *MyD88*^*+/+*^ and *MyD88*^*-/-*^ *IL-12p40 eYFP* lamina propria myeloid cell populations. (A) Dendritic cells were defined as MHCII^+^ CD11c^+^ F4/80^-^, (B) neutrophils were identified as Ly6G^+^, (C) macrophages were defined as F4/80^+^. Numbers show the percent of cells falling within the indicated gates. This figure shows the results from one representative noninfected *MyD88*^*+/+*^ mouse.
(TIF)

S6 Fig Mesenteric lymph node IL-12p40 is produced independently of MyD88 during oral infection. Mice were infected and tissues harvested as described in Fig 2 legend. (A) Total number of DC, PMN and MAC in mesenteric lymph nodes (MLN) from MyD88 WT and KO mice. (B) Flow cytometric analysis of IL-12p40 expression in the presence and absence of MyD88. Numbers in the scatter plots indicate the percent of cells falling within the indicated gate. (C) Frequency and (D) number of IL-12 positive cells amongst DC, PMN and MAC in the MLN. (E) Frequency of IL-12p40 expression amongst mesenteric lymph node DC subsets. (F) IL-12p40 secretion in bulk cultures of MLN cells cultured in media or stimulated with STAg. The solid line indicates IL-12p40 levels produced by noninfected KO MLN cells. Values are the means ± SEM of two independent experiments and each symbol represents an individual mouse. Open and closed symbols delineate data obtained from one independent experiment. The data were analyzed in an un-paired Student’s *t* test where * p<0.05.
(TIF)

S7 Fig Lymphocyte populations in lamina propria and mesenteric lymph nodes of infected *MyD88*^*+/+*^ and *MyD88*^*-/-*^ mice. LP and MLN cells were collected from day 7 infected mice and analyzed by flow cytometry. Frequencies of CD4^+^ and CD8^+^ lymphocytes within the TCR-β^+^ gate were determined in the (A) LP and (B) MLN. Values are the means ± SEM of two independent experiments (n = 8/group).
(TIF)

S8 Fig *T*. *gondii* triggers a strong, MyD88-independent IFN-γ response in the mesenteric lymph nodes. MLN cells were collected from *MyD88*^*+/+*^ and *MyD88*^*-/-*^ mice one week after infection and flow cytometry was used to assess IFN-γ production by T cells. (A) Representative IFN-γ expression by CD4^+^ and CD8^+^ T cells from naïve and infected mice. The frequencies of IFN-γ^+^ cells from multiple mice are shown in B. (C) Mean fluorescence intensity of IFN-γ^+^ CD4^+^ and CD8^+^ T cell populations in the MLN Flow cytometry was repeated independently with similar results. (D) IFN-γ secretion by MLN cells cultured *ex vivo* from two independent experiments. Background levels of IFN-γ released by cells from noninfected WT and KO mice were below 400 pg/ml. Each individual mouse is represented by a symbol. Unpaired Student’s *t* test (D) and one-way ANOVA with Tukey multiple comparisons post-test (B) was used to analyze the data where * p<0.05 **p<0.01 ***p<0.001.
(TIF)

S9 Fig Assessment of MHCII tetramer binding specificity. Small intestine LP cells and MLN cells were isolated from WT and KO mice at day 7 post-infection. (A) Live T cells were defined as zombie aqua negative, TCR-β+, and CD4^+^ or CD8^+^. (B) *Toxoplasma* (AS15) and flagellin (CBir1) MHCII tetramer staining was assessed on CD8^+^ T lymphocytes as a control for specificity. (C) AS15 and CBir1 tetramer^+^ CD8^+^ T cells in the LP and MLN of *MyD88*^*+/+*^ and *MyD88*^*-/-*^ mice.
(TIF)

S10 Fig Innate lymphoid cell populations in the lamina propria of naïve *MyD88*^*+/+*^ and *MyD88*^*-/-*^ mice. Small intestine LP cells were isolated from naïve mice and analyzed by flow cytometry. (A) ILC were classified as Lineage negative (CD3^-^, Ly-6G/Ly-6C^-^, CD11b^-^, CD45R/B220^-^, and TER-119^-^) CD90^hi^ and CD127^+^ or CD127^-^. (B) The CD127^+^ ILC population was further gated to characterize T-bet^+^ ILC1, GATA3^+^ ILC2, or RORγt^+^ ILC3. (C) The CD127^-^ ILC population was gated similarly to determine ILC1, ILC2, and ILC3 populations. The frequencies and cell numbers of (D, F) CD127^+^ ILC, ILC1, ILC2, and ILC3 and (E, G) CD127^-^ ILC1, ILC2 and ILC3 were calculated and graphed. Open and closed symbols indicate individual mice from two independent experiments. Unpaired Student’s *t* test, where *p<0.05.
(TIF)

S11 Fig TNF-α is not required for generating mucosal anti-Toxoplasma immunity in the absence of MyD88. (A) TNF-α depletions and isotype injections were performed according to the schematic. Tissues were harvested at day 7 post-infection and LP cells were cultured for 72 hours with and without STAg. (A) IL-12 and (B) IFN-γ levels were quantified by ELISA. n = 12/group. Values are the means ± SEM of three independent experiments.
(TIF)
