# Supplementary figures and images for: Induction of IL-12p40 and type 1 immunity by Toxoplasma gondii in the absence of the TLR-MyD88 signaling cascade
Source: PLoS Pathog. 2021 Oct 1;17(10):e1009970. doi: 10.1371/journal.ppat.1009970 (PMC8513874; doi:10.1371/journal.ppat.1009970)

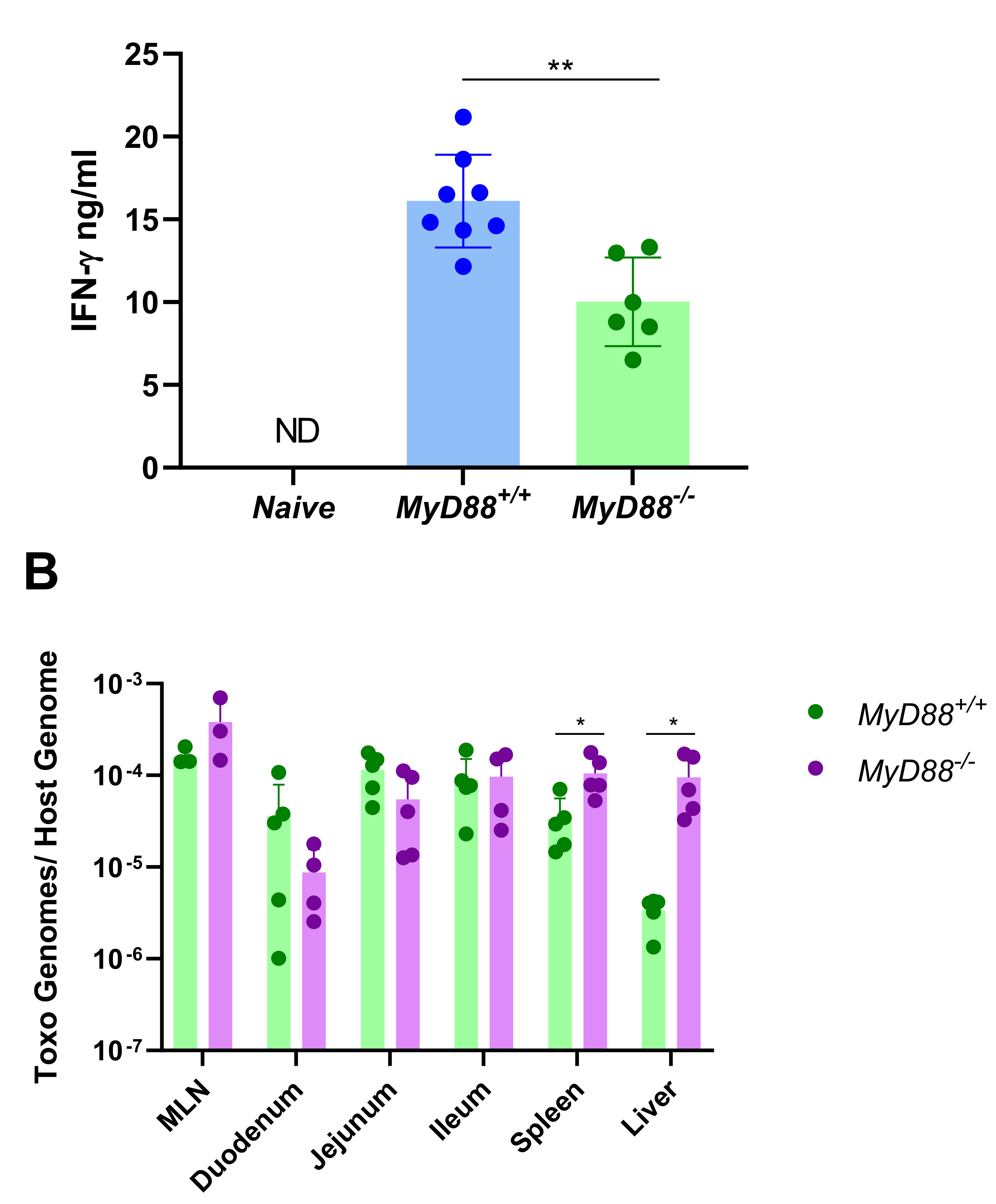

Supplement: S1 Fig — MyD88+/+ and MyD88-/- mice were orally inoculated with 40 ME49 cysts. (A) One-week after infection, serum IFN-γ levels were measured by ELISA (n = 6-8/genotype). Naïve WT and KO serum IFN-γ (n = 3-4/genotype) was not detected in the ELISA (ND). (B) Duodenum, jejunum, ileum, spleen, liver, and MLN tissues were also collected from mice at day 7 post-infection. Parasite burden was quantified using tissues from mice that were orally infected with 40 cysts. Shown is a representative experiment of 4 performed. An unpaired Student’s t test was used to compare infected WT and KO mice where *p<0.05 **p<0.01 ***p<0.001. (TIF) [file ppat.1009970.s001.tif]

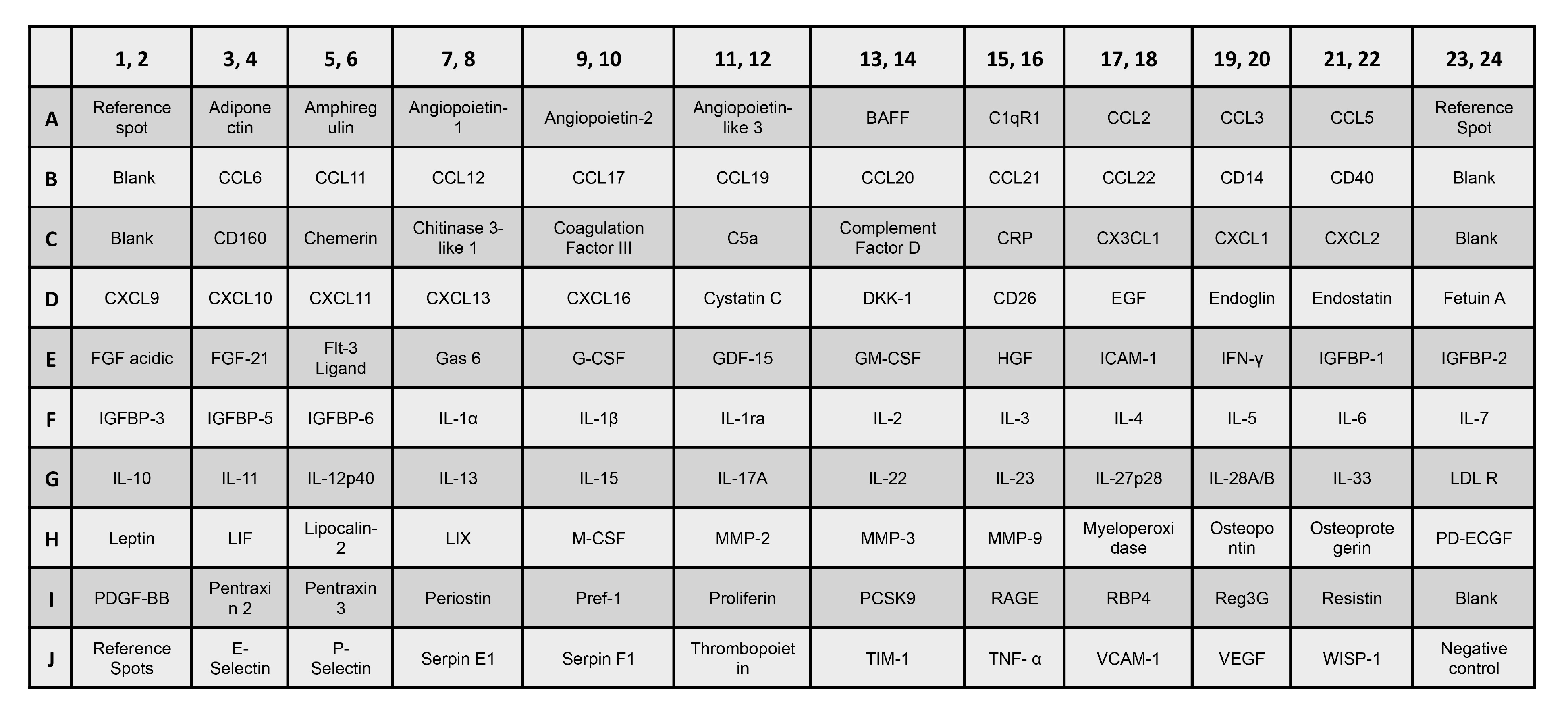

Supplement: S2 Fig — (TIF) [file ppat.1009970.s002.tif]

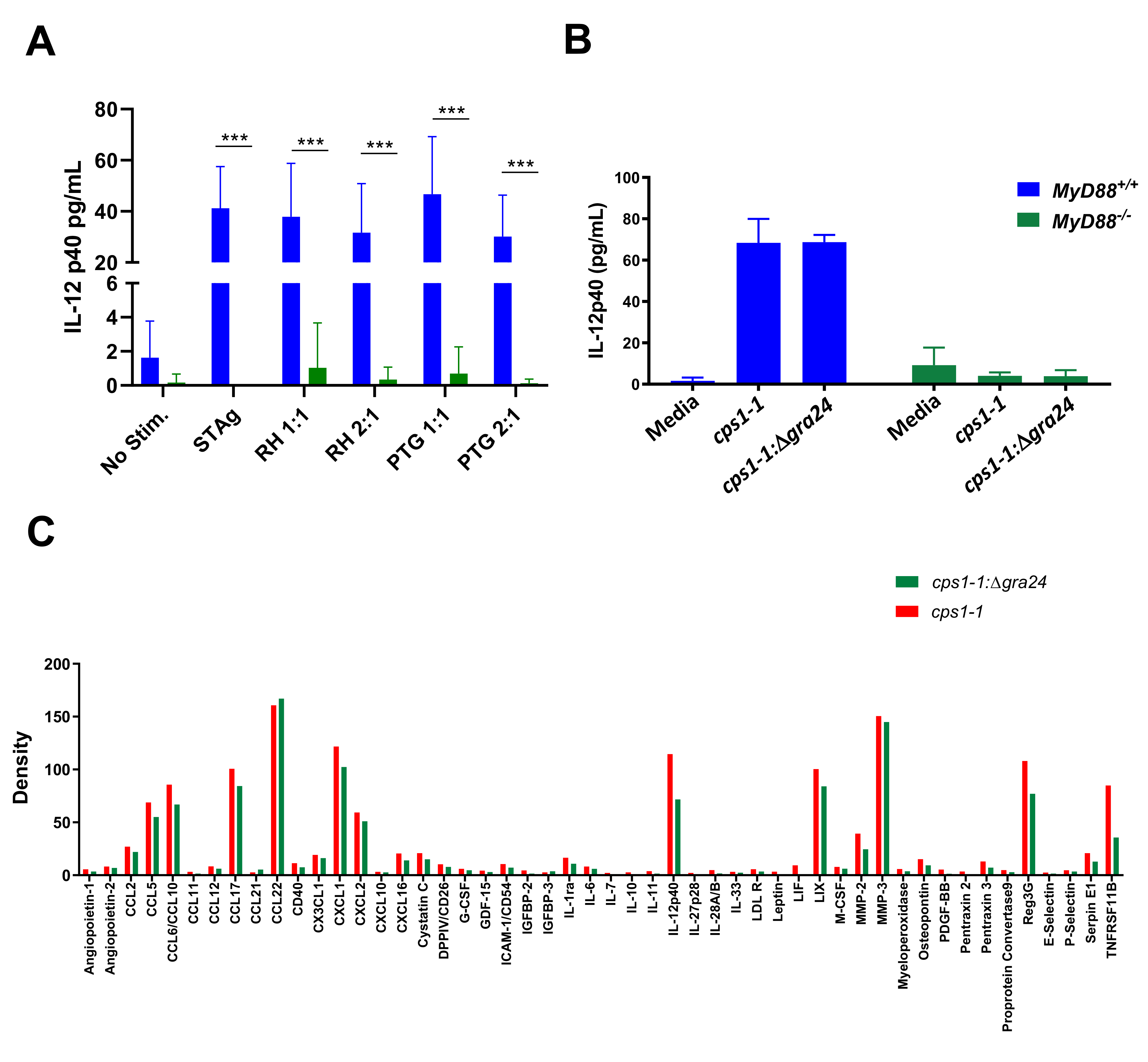

Supplement: S3 Fig — (A) Naïve LP cells were cultured ex vivo with STAg or infected with a type I strain (RH) or type II strain (PTG) of T. gondii at the indicated MOI. Supernatants were collected after 72 hrs and IL-12p40 was quantified by ELISA. Values are the means ± SEM of three independent experiments. (B) Similarly, supernatants from cps1-1 and cps1-1Δgra24 infected MyD88+/+ and MyD88-/- LP cells were collected to quantify IL-12p40 production by ELISA. (C) Naïve WT LP cells were infected with cps1-1 or cps1-1Δgra24 tachyzoites (MOI 1:1) and supernatants were harvested at 72 hours to analyze secreted immune factors using a mouse cytokine/chemokine proteome array. An unpaired Student’s t test was used to compare infected WT and KO responses where ***p<0.001. (TIF) [file ppat.1009970.s003.tif]

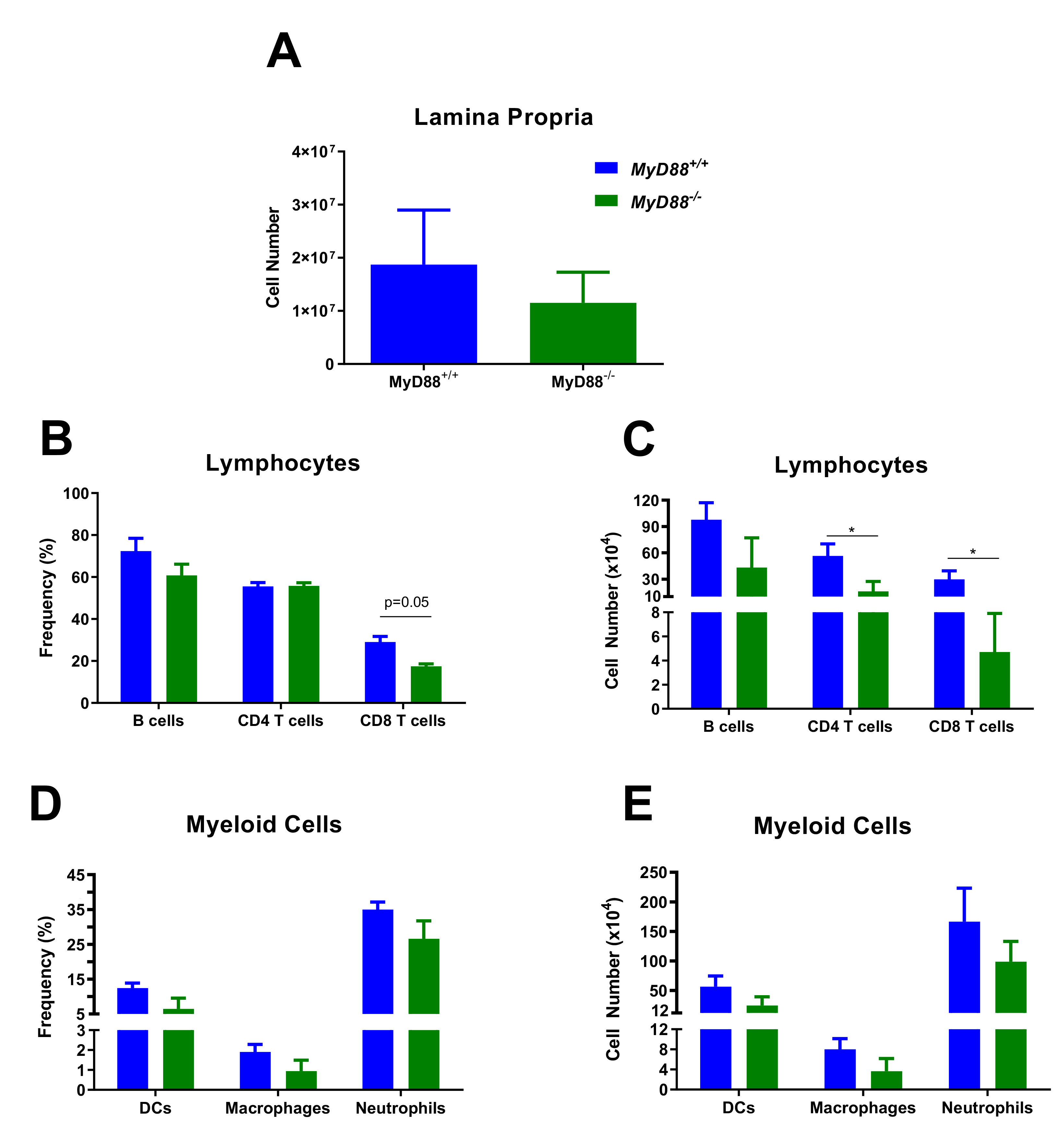

Supplement: S4 Fig — Naïve mice were euthanized for tissue collection. (A) Total LP cell counts. Flow cytometry was used to determine frequencies of lymphocytes and myeloid cells (B and D, respectively), and total numbers of lymphocytes and myeloid cells were subsequently calculated (C and E, respectively). Values are the means ± SEM of three independent experiments with a total of n = 9 mice/genotype. Unpaired Student’s t test used to compare genotypes where *p<0.05. (TIF) [file ppat.1009970.s004.tif]

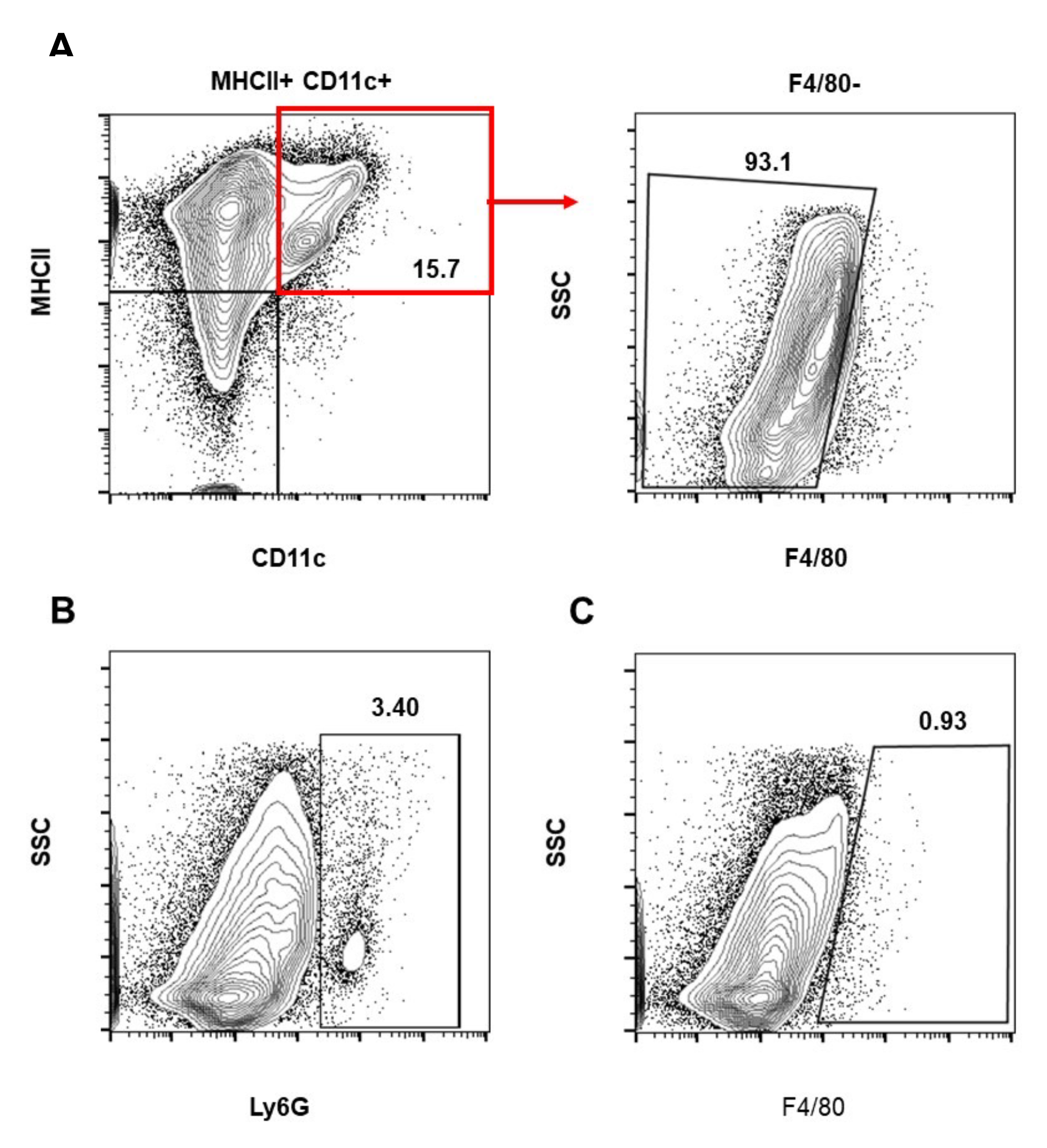

Supplement: S5 Fig — (A) Dendritic cells were defined as MHCII+ CD11c+ F4/80-, (B) neutrophils were identified as Ly6G+, (C) macrophages were defined as F4/80+. Numbers show the percent of cells falling within the indicated gates. This figure shows the results from one representative noninfected MyD88+/+ mouse. (TIF) [file ppat.1009970.s005.tif]

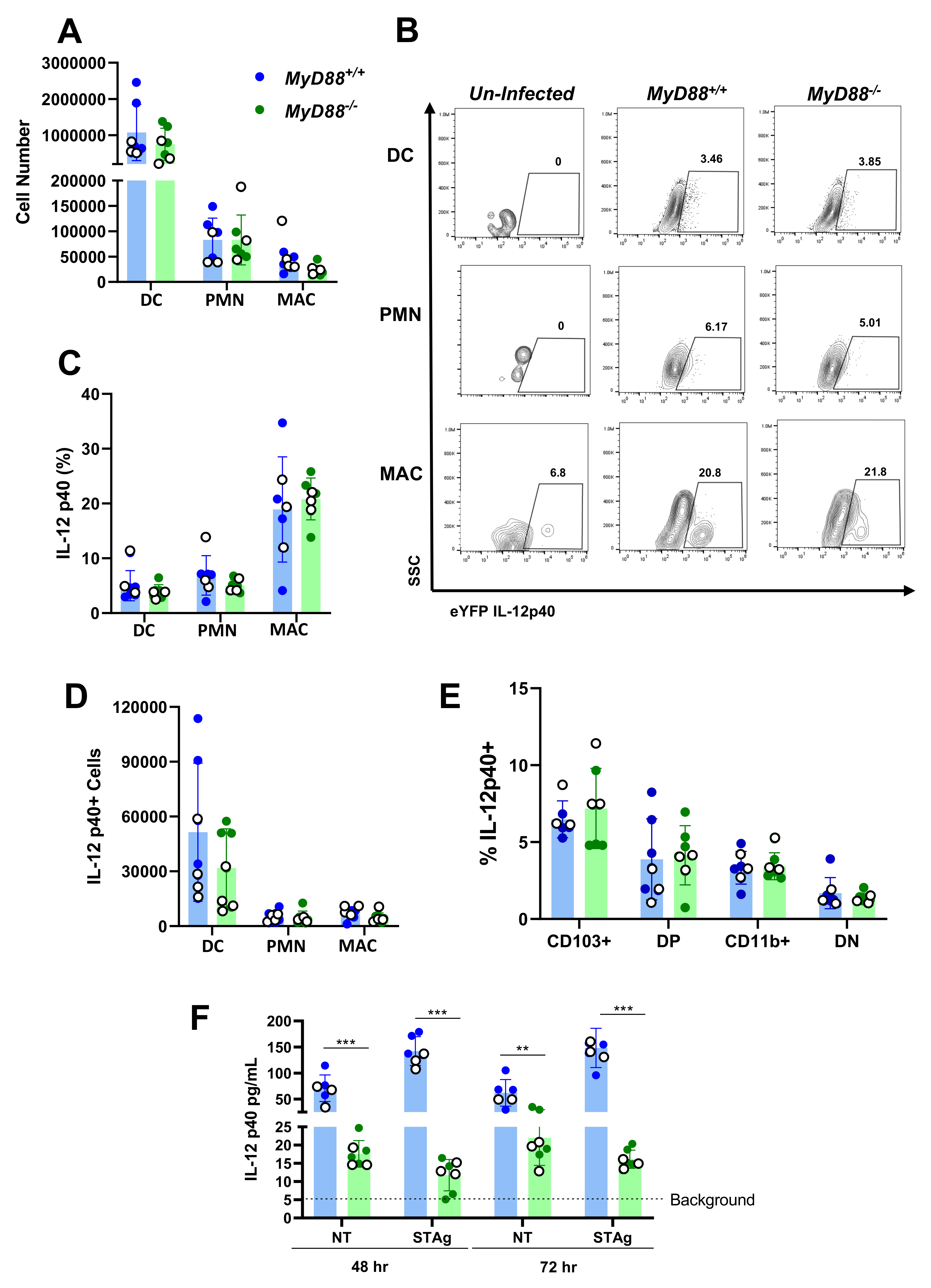

Supplement: S6 Fig — Mice were infected and tissues harvested as described in Fig 2 legend. (A) Total number of DC, PMN and MAC in mesenteric lymph nodes (MLN) from MyD88 WT and KO mice. (B) Flow cytometric analysis of IL-12p40 expression in the presence and absence of MyD88. Numbers in the scatter plots indicate the percent of cells falling within the indicated gate. (C) Frequency and (D) number of IL-12 positive cells amongst DC, PMN and MAC in the MLN. (E) Frequency of IL-12p40 expression amongst mesenteric lymph node DC subsets. (F) IL-12p40 secretion in bulk cultures of MLN cells cultured in media or stimulated with STAg. The solid line indicates IL-12p40 levels produced by noninfected KO MLN cells. Values are the means ± SEM of two independent experiments and each symbol represents an individual mouse. Open and closed symbols delineate data obtained from one independent experiment. The data were analyzed in an un-paired Student’s t test where * p<0.05. (TIF) [file ppat.1009970.s006.tif]

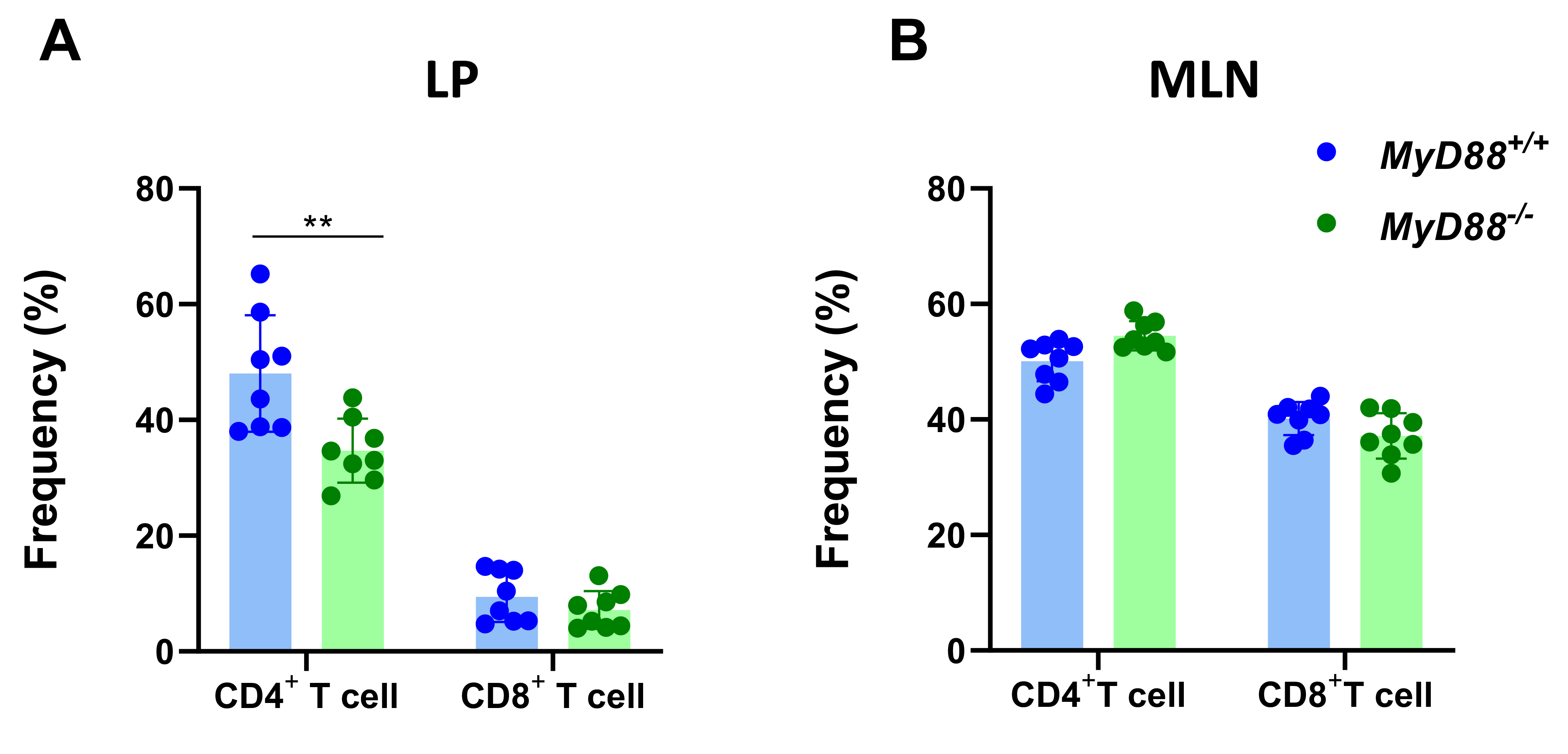

Supplement: S7 Fig — LP and MLN cells were collected from day 7 infected mice and analyzed by flow cytometry. Frequencies of CD4+ and CD8+ lymphocytes within the TCR-β+ gate were determined in the (A) LP and (B) MLN. Values are the means ± SEM of two independent experiments (n = 8/group). (TIF) [file ppat.1009970.s007.tif]

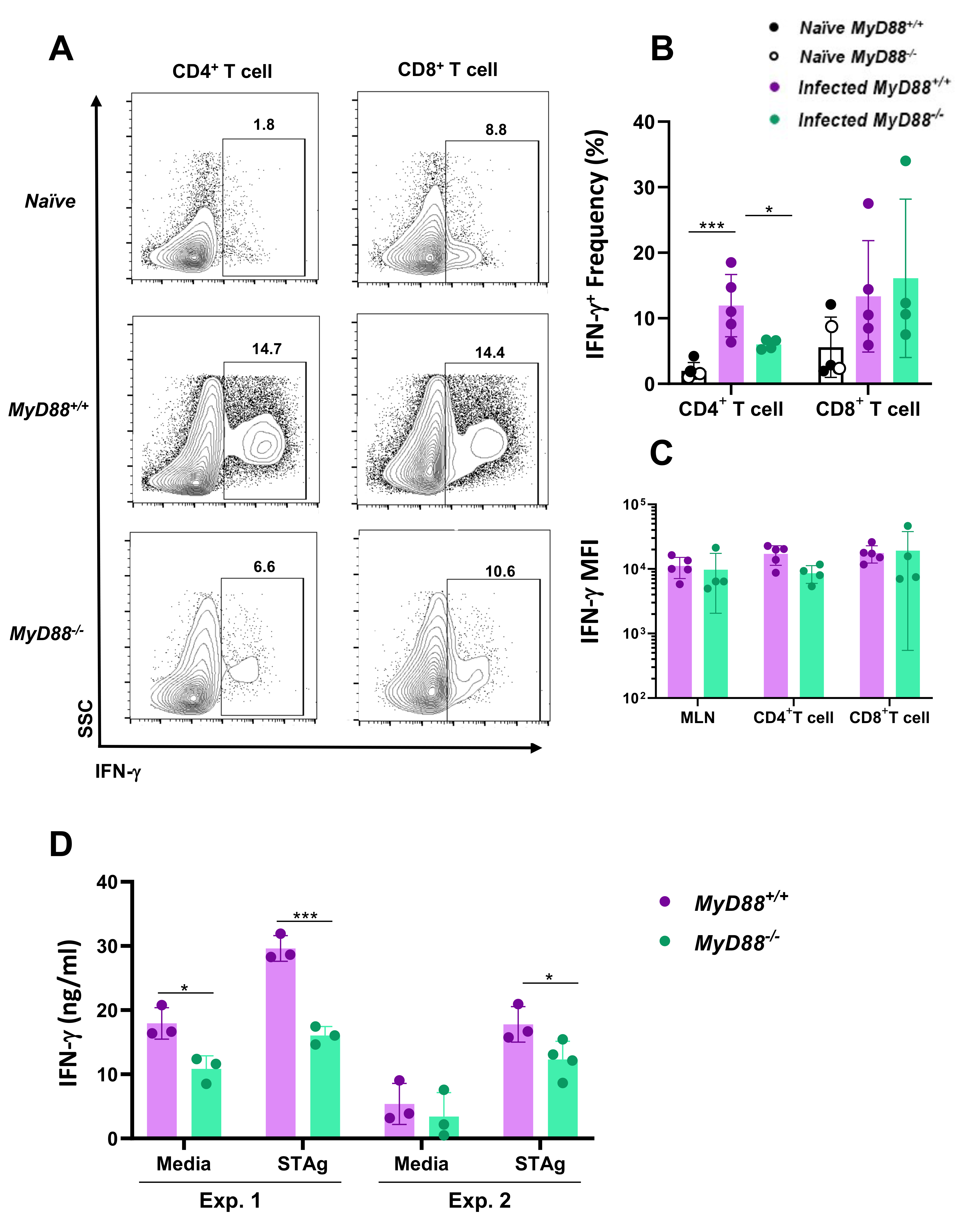

Supplement: S8 Fig — MLN cells were collected from MyD88+/+ and MyD88-/- mice one week after infection and flow cytometry was used to assess IFN-γ production by T cells. (A) Representative IFN-γ expression by CD4+ and CD8+ T cells from naïve and infected mice. The frequencies of IFN-γ+ cells from multiple mice are shown in B. (C) Mean fluorescence intensity of IFN-γ+ CD4+ and CD8+ T cell populations in the MLN Flow cytometry was repeated independently with similar results. (D) IFN-γ secretion by MLN cells cultured ex vivo from two independent experiments. Background levels of IFN-γ released by cells from noninfected WT and KO mice were below 400 pg/ml. Each individual mouse is represented by a symbol. Unpaired Student’s t test (D) and one-way ANOVA with Tukey multiple comparisons post-test (B) was used to analyze the data where * p<0.05 **p<0.01 ***p<0.001. (TIF) [file ppat.1009970.s008.tif]

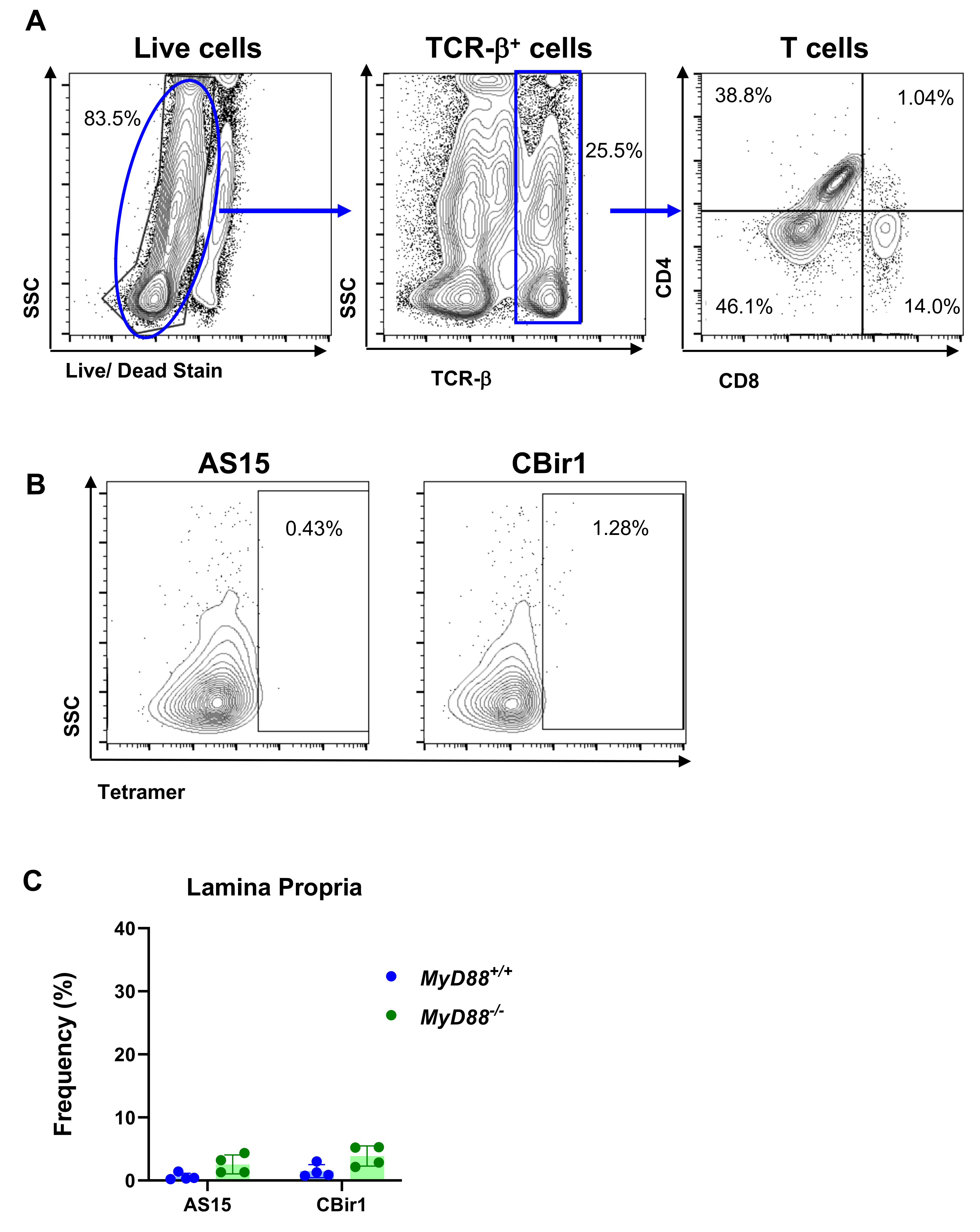

Supplement: S9 Fig — Small intestine LP cells and MLN cells were isolated from WT and KO mice at day 7 post-infection. (A) Live T cells were defined as zombie aqua negative, TCR-β+, and CD4+ or CD8+. (B) Toxoplasma (AS15) and flagellin (CBir1) MHCII tetramer staining was assessed on CD8+ T lymphocytes as a control for specificity. (C) AS15 and CBir1 tetramer+ CD8+ T cells in the LP and MLN of MyD88+/+ and MyD88-/- mice. (TIF) [file ppat.1009970.s009.tif]

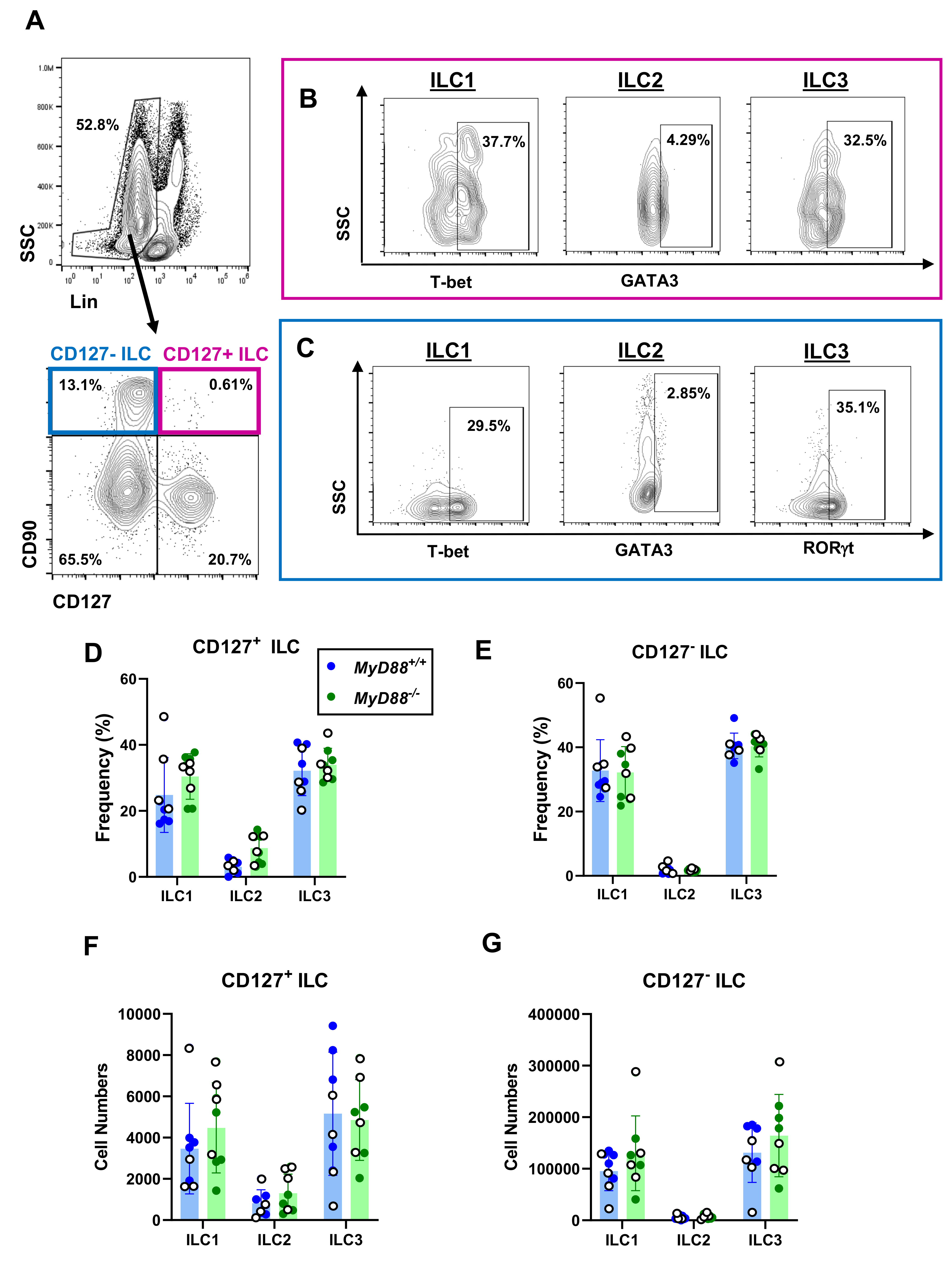

Supplement: S10 Fig — Small intestine LP cells were isolated from naïve mice and analyzed by flow cytometry. (A) ILC were classified as Lineage negative (CD3-, Ly-6G/Ly-6C-, CD11b-, CD45R/B220-, and TER-119-) CD90hi and CD127+ or CD127-. (B) The CD127+ ILC population was further gated to characterize T-bet+ ILC1, GATA3+ ILC2, or RORγt+ ILC3. (C) The CD127- ILC population was gated similarly to determine ILC1, ILC2, and ILC3 populations. The frequencies and cell numbers of (D, F) CD127+ ILC, ILC1, ILC2, and ILC3 and (E, G) CD127- ILC1, ILC2 and ILC3 were calculated and graphed. Open and closed symbols indicate individual mice from two independent experiments. Unpaired Student’s t test, where *p<0.05. (TIF) [file ppat.1009970.s010.tif]

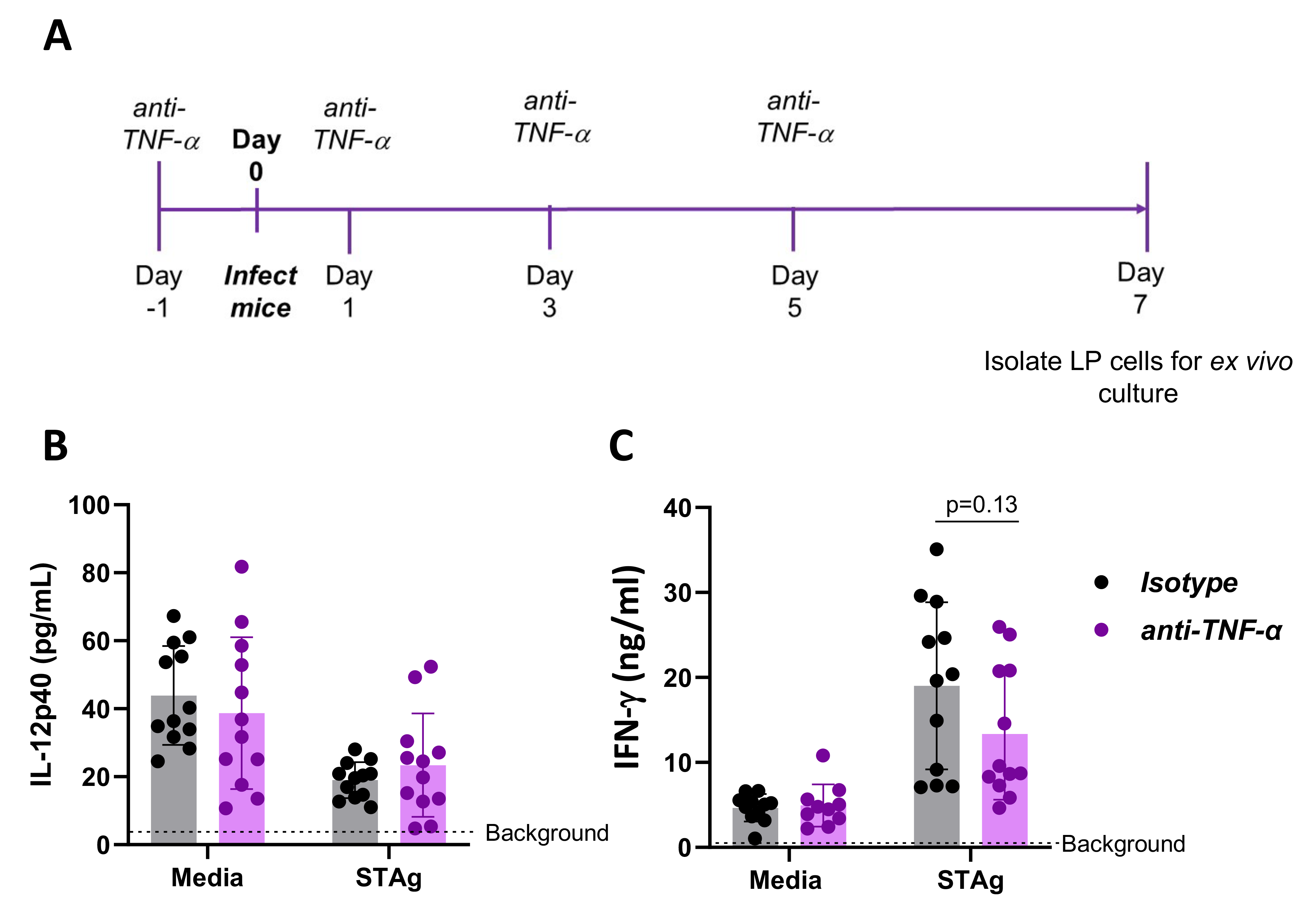

Supplement: S11 Fig — (A) TNF-α depletions and isotype injections were performed according to the schematic. Tissues were harvested at day 7 post-infection and LP cells were cultured for 72 hours with and without STAg. (A) IL-12 and (B) IFN-γ levels were quantified by ELISA. n = 12/group. Values are the means ± SEM of three independent experiments. (TIF) [file ppat.1009970.s011.tif]
